# Pyrazine synthesis: a century-long evolution from condensation to modern catalytic strategies

**DOI:** 10.1039/d6ra05539g

**Published:** 2026-09-18

**Authors:** Bingyang Wang, Yao Zhao, Weihua Chen, Lianchen Zhao, Qian Yang, Jie Ren, Guanglu Wang, Xiaoru Wang

**Affiliations:** a School of Tobacco Science and Engineering, Zhengzhou University of Light Industry No. 136 Ke Xue Avenue Zhengzhou Henan 450002 People's Republic of China 2021038@zzuli.edu.cn; b China Tobacco Hebei Industrial Co., Ltd Shijiazhuang Hebei 050000 People's Republic of China 13260648079@163.com

## Abstract

Pyrazine (1,4-diazabenzene) is a privileged nitrogen-containing heterocycle of considerable importance in pharmaceuticals, food flavourings, agrochemicals, and functional materials. Since its first deliberate synthesis in 1908, the methods for pyrazine construction have undergone a remarkable evolution spanning more than a century. This review provides a comprehensive and critically evaluative account of that journey, tracing the development from early classical condensation reactions—namely the α-aminoketone self-condensation (Tutin reaction) and the α-diketone–α-diamine condensation–oxidation—through industrial heterogeneous catalytic systems (copper-chromite, zinc-chromite, and platinum-based catalysts), to the precision functionalisation afforded by palladium-catalysed cross-coupling, and ultimately to the green and sustainable strategies that have emerged over the past fifteen years, including solvent-free, metal-free, electrochemical, amino-acid-based, and biomass-derived methodologies. Distinct from earlier accounts, this review is organised by methodological strategy, with each approach critically assessed for its scope, limitations, and efficiency. The evolutionary logic from conditional construction to precision modification and, finally, to green sustainability is elucidated, together with an outlook on future directions encompassing earth-abundant metal catalysis, biomass feedstock conversion, and emerging applications in optoelectronic and framework materials. By systematically integrating historical perspective with contemporary green chemistry principles, this review serves both as a practical reference for synthetic chemists and as a roadmap for future methodological innovation in pyrazine synthesis.

## Abstract 2

This review charts the century-long evolution of pyrazine synthesis, from classical condensation to modern sustainable approaches, with critical assessment of each methodological strategy.

## Graphical abstract

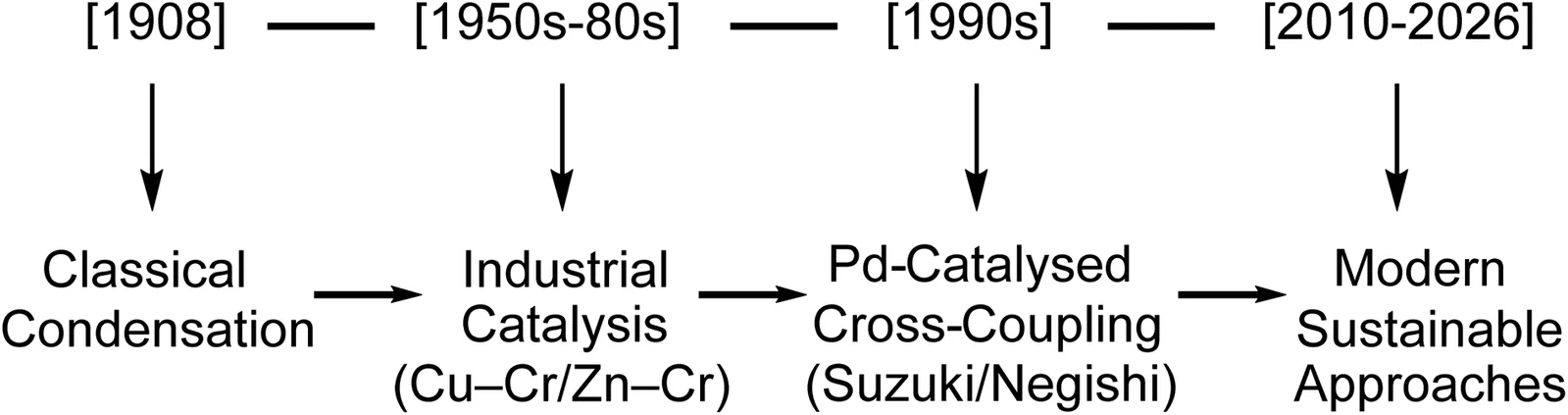

## 1. Introduction

Pyrazine (1,4-diazabenzene) is a privileged nitrogen-containing heterocycle that occurs widely in natural products, pharmaceuticals, food flavourings, and functional materials. Its distinctive aromaticity and strong coordinating ability underpin its indispensable role across medicine, agrochemicals, dyes, food additives, and metal–organic frameworks.

The systematic study of pyrazine chemistry commenced in 1876 with the Staedel–Rügheimer synthesis, and was subsequently advanced by Gutknecht's refinements and Mason's structural investigations of ‘piazine’ derivatives.^1–4^ The first deliberate synthesis of a pyrazine derivative—2,5-diphenylpyrazine—was reported by Gabriel in 1908.^5^ Over more than a century, pyrazine synthesis has evolved from classical condensation reactions into modern green catalytic strategies.

Early synthetic routes relied predominantly on the self-condensation of α-aminoketones and on the condensation and oxidation of α-diketones with α-diamines. The late 20th century saw the introduction of homogeneous and heterogeneous catalytic systems, with copper-chromite and zinc-chromite catalysts enabling industrial vapour-phase dehydrogenative cyclization. The 1990s witnessed the advent of palladium-catalysed cross-coupling reactions, which ushered in an era of precise pyrazine functionalisation. In the 21st century, guided by the principles of green chemistry, a diverse array of methods—solvent-free, metal-free, electrochemical, heterogeneous catalytic, and biomass-derived—have dramatically expanded the synthetic toolbox.

Despite the long history and rich diversity of pyrazine synthetic methodologies, a comprehensive, critically evaluative overview that systematically compares the strengths and limitations of the various approaches from classical condensations to modern green catalysis remains absent. Existing reviews on pyrazine chemistry have largely concentrated on specific reaction types (*e.g.*, cross-coupling, metal-free multicomponent reactions, or heterogeneous catalysis) or have been organised by synthetic method without systematic critical comparison of different strategies, offering limited insight into the methodological evolution and its underlying driving forces (Table 1).^6–11^ The past decade has witnessed an unprecedented expansion of green and sustainable synthetic methods including solvent-free protocols, electrochemical synthesis, biomass-derived routes, and natural product catalysis. Yet these advances have not been systematically integrated into a unified framework that traces the evolutionary logic from conditional construction to precision modification and, ultimately, to green sustainability. Moreover, the escalating demand for pyrazine-based pharmaceuticals, agrochemicals, and functional materials has created a pressing need for synthetic chemists to select the most appropriate method for a given target, a choice that requires a clear understanding of the advantages, limitations, and scope of each approach. This review addresses these gaps by providing: (i) a methodological strategy-based organisation that moves beyond purely chronological accounts; (ii) critical assessments of each method's scope and limitations; and (iii) an integrated perspective on how pyrazine synthesis has evolved in response to the changing demands of industry, pharmaceutical discovery, and green chemistry principles.

**Table 1:** Comparison of the present review with major existing reviews on pyrazine chemistry

Review	Year	Coverage period	Organizational principle	Industrial catalysis covered?	Critical comparison of methods?	Evolutionary logic?
Higasio & Shoji (ref. 7)	2001	Pre-2000	By compound type (pyridines, pyrazines, *etc.*)	Yes	No	No
Nikishkin *et al.* (ref. 8)	2013	1990s–2013	By cross-coupling reaction type	No	Partial	No
Ong *et al.* (ref. 72)	2017	1944–2015	By synthetic method	Mentioned	No	No
Mortzfeld *et al.* (ref. 6)	2020	Pre-2020	By production strategy (chemical/enzymatic/fermentation)	Mentioned	No	No
Preeti & Singh (ref. 10)	2021	2000–2021	By reaction type (MCRs) for six-membered N-heterocycles	No	Yes (within MCRs)	No
Choudhary *et al.* (ref. 11)	2023	2015–2021	By reaction type (intra-*vs.* intermolecular)	No	No	No
This work	2026	1908–2026	By methodological strategy & evolutionary logic	Yes	Yes	Yes

In this review, ‘pyrazine synthesis’ is defined in a broad sense, encompassing both *de novo* construction of the pyrazine ring from acyclic precursors and late-stage functionalization of preformed pyrazine scaffolds. The historical discussion is included not merely for chronological completeness, but to illustrate how early discoveries, despite their practical limitations, established the fundamental reaction patterns and regioselectivity challenges that later shaped the development of modern catalytic methods. The literature covered in this review was selected to highlight methods that represent distinct conceptual or strategic advances in pyrazine synthesis, rather than routine substrate extensions, with emphasis on broadly cited studies and recent green chemistry developments. The literature was surveyed using Web of Science, Sci-Finder with relevant search terms. We have prioritised representative examples that best illustrate the scope, limitations, and evolutionary logic of each methodological class. Coverage extends to early 2026 for key developments.

This review is organised by methodological strategy to highlight the evolutionary logic from conditional construction to precision modification and, ultimately, to green sustainability. Section 2 covers the classical condensation–oxidation routes and provides a critical assessment of their limitations. Section 3 discusses industrial heterogeneous catalysis. Section 4 focuses on palladium-catalysed cross-coupling for regioselective functionalisation. Section 5 surveys green innovations, including solvent-free, metal-free, electrochemical, amino-acid-based approaches, together with selected examples of heterogeneous catalysis and continuous-flow processing; emerging photocatalytic strategies are discussed in the outlook (Section 6).

## 2. Classical methods: condensation and oxidation

Before 1980, two classical routes dominated pyrazine synthesis: the self-condensation of α-aminoketones (Tutin reaction) and the condensation–oxidation of α-diketones with α-diamines.^5,12^ The former, although mechanistically informative, suffers from poor regiocontrol and typically affords mixtures of isomers; the latter reliably delivers 2,3-disubstituted pyrazines but requires a dedicated oxidation step. This section provides a critical evaluation of both strategies (Sections 2.1 and 2.2), then discusses further transformations of dihydropyrazine intermediates (Section 2.3) and additional homogeneous condensation routes developed in the 1980s (Section 2.4), before concludes with a comparative summary (Section 2.5).

### 2.1 α-Aminoketone self-condensation (Tutin reaction)

**Scheme 1:**
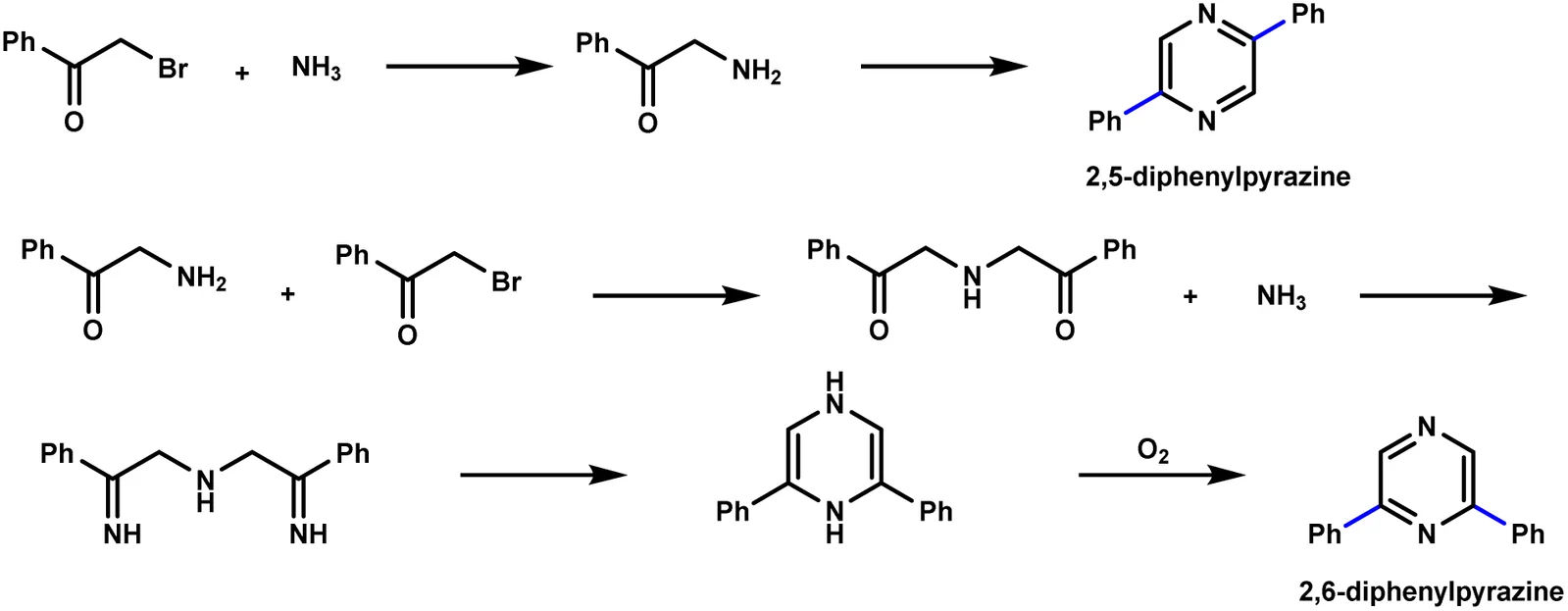
Formation of 2,5- and 2,6-diphenylpyrazine in Gabriel–Tutin reaction.

The α-aminoketone self-condensation represents the earliest established method for pyrazine synthesis. In 1908, Gabriel reported the formation of 2,5-diphenylpyrazine from ω-bromoacetophenone and ammonia.^5^ In 1910, Tutin corrected this finding by demonstrating that both 2,5- and 2,6-diphenylpyrazines were produced in nearly equimolar amounts, and proposed that the 2,6-isomer arises *via* a di(benzoylmethyl)amine intermediate (Scheme 1).^12^ Subsequently, Cornforth extended the method to alkyl systems, obtaining 2,5-dimethylpyrazine from aminoacetone.^13^

**Scheme 2:**
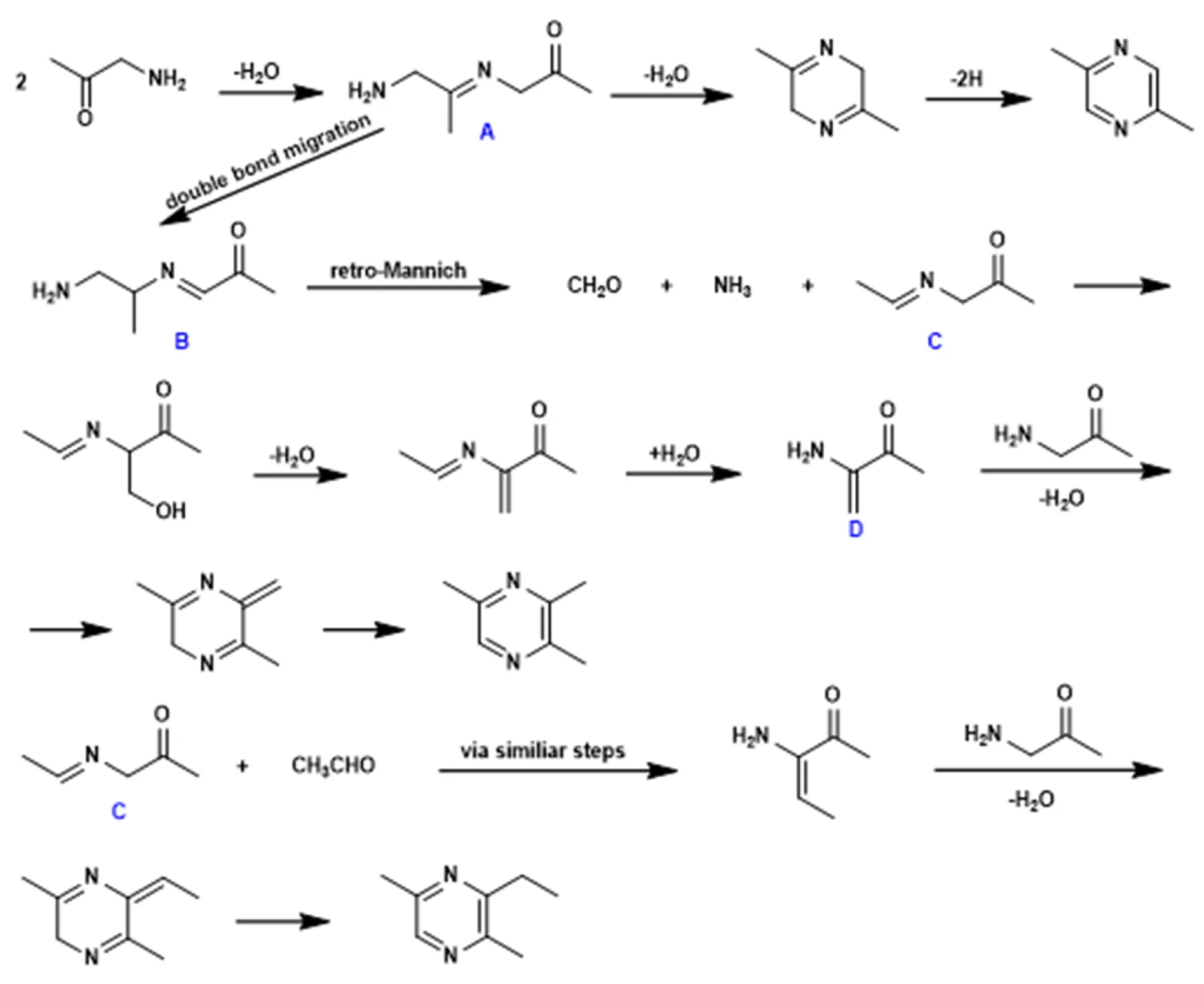
Retro Mannich fragmentation leading to polyalkylpyrazines.

A significant mechanistic insight was provided by Rizzi, who demonstrated that the self-condensation of aminoacetone yields not only 2,5-dimethylpyrazine but also trimethylpyrazine and ethyl-dimethylpyrazine in an approximate ratio of 50 : 33 : 17.^14^ He proposed that the dihydropyrazine intermediate undergoes retro-Mannich fragmentation, liberating small aldehydes (formaldehyde, acetaldehyde) which then re-enter the condensation to append additional methyl or ethyl groups onto the pyrazine ring (Scheme 2). The addition of exogenous formaldehyde or acetaldehyde markedly increased the yields of polyalkylpyrazines, thereby confirming this mechanistic pathway.

The α-aminoketone self-condensation provides a straightforward route to symmetric 2,5-diaryl- and dialkylpyrazines and has yielded valuable mechanistic insights into polyalkylation. However, the method suffers from several inherent limitations. α-Aminoketones are frequently unstable and prone to side reactions. When unsymmetrical substrates are employed, the reaction typically affords nearly inseparable mixtures of 2,5- and 2,6-isomers. Although mechanistically intriguing, the retro-Mannich fragmentation complicates the product distribution, and typical isolated yields of unsymmetrical pyrazines remain low, often below 40%. Consequently, the industrial applicability of this approach is severely limited by the difficulties in isomer separation and its poor atom economy.

### 2.2 α-Diketone–α-diamine condensation–oxidation

**Scheme 3:**
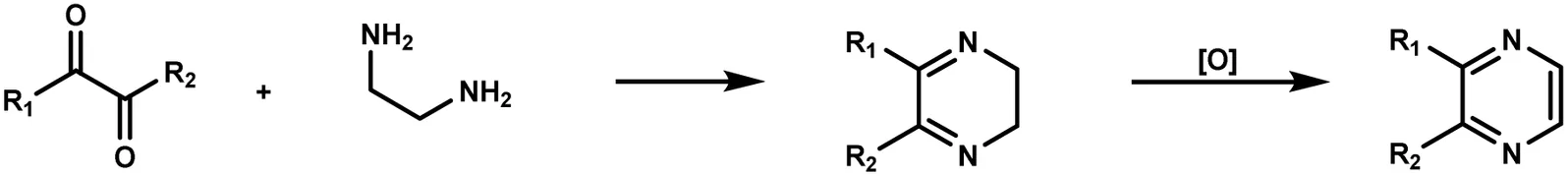
Classical condensation–oxidation route to pyrazines.

This route proceeds *via* the initial condensation of an α-diketone with an α-diamine to afford a 2,3-dihydropyrazine intermediate, which is then converted into the target pyrazine through dehydrogenative aromatisation (Scheme 3). The methodology is thoroughly documented in authoritative treatises and is consistently regarded as a classical approach in modern reviews.^6,15,16^

In 1958, Ishiguro and Matsumura first reported the condensation of butane-2,3-dione with ethylenediamine in diethyl ether at room temperature, obtaining 2,3-dimethyl-5,6-dihydropyrazine in 85% yield.^17^ Subsequent refluxing of this intermediate in KOH/ethanol afforded 2,3-dimethylpyrazine. Although operationally straightforward, this dehydrogenation step frequently left residual dihydropyrazine, which led to undesirable colour and odour instability in the products upon storage. In 1967, Flament and Stoll systematically extended this method to a range of 2,3-dioxoalkanes.^18^ By employing unsymmetrical diketones, they obtained 2-methyl-3-alkyl-5,6-dihydropyrazines in 35–75% yield with high regioselectivity; subsequent catalytic dehydrogenation over copper chromite at 300–350 °C under a nitrogen stream furnished the corresponding 2-methyl-3-alkylpyrazines in 55–100% overall yield. Despite the high yields, this procedure necessitated elevated temperatures and specialised catalytic apparatus.

A significant advance in the dehydrogenation step was reported by Akiyama and co-workers in 1978.^19^ Building on Ishiguro's KOH/ethanol system, they introduced MnO_2_ or CuO as oxidants, thereby achieving efficient dehydrogenation of dihydropyrazines under reflux. Using an optimised molar ratio of dihydropyrazine/KOH/metal oxide = 1 : 1 : 3, alkylpyrazines were obtained in nearly 80% yield with >99% purity and were free of residual dihydropyrazine. These products exhibited long-term storage stability without discolouration or off-odours, thereby satisfying the stringent purity and stability criteria demanded for food flavourings. Notably, Akiyama's use of stoichiometric MnO_2_ presaged later tandem oxidation strategies that obviate the need to isolate labile 1,2-dicarbonyl intermediates.^20,21^

**Scheme 4:**
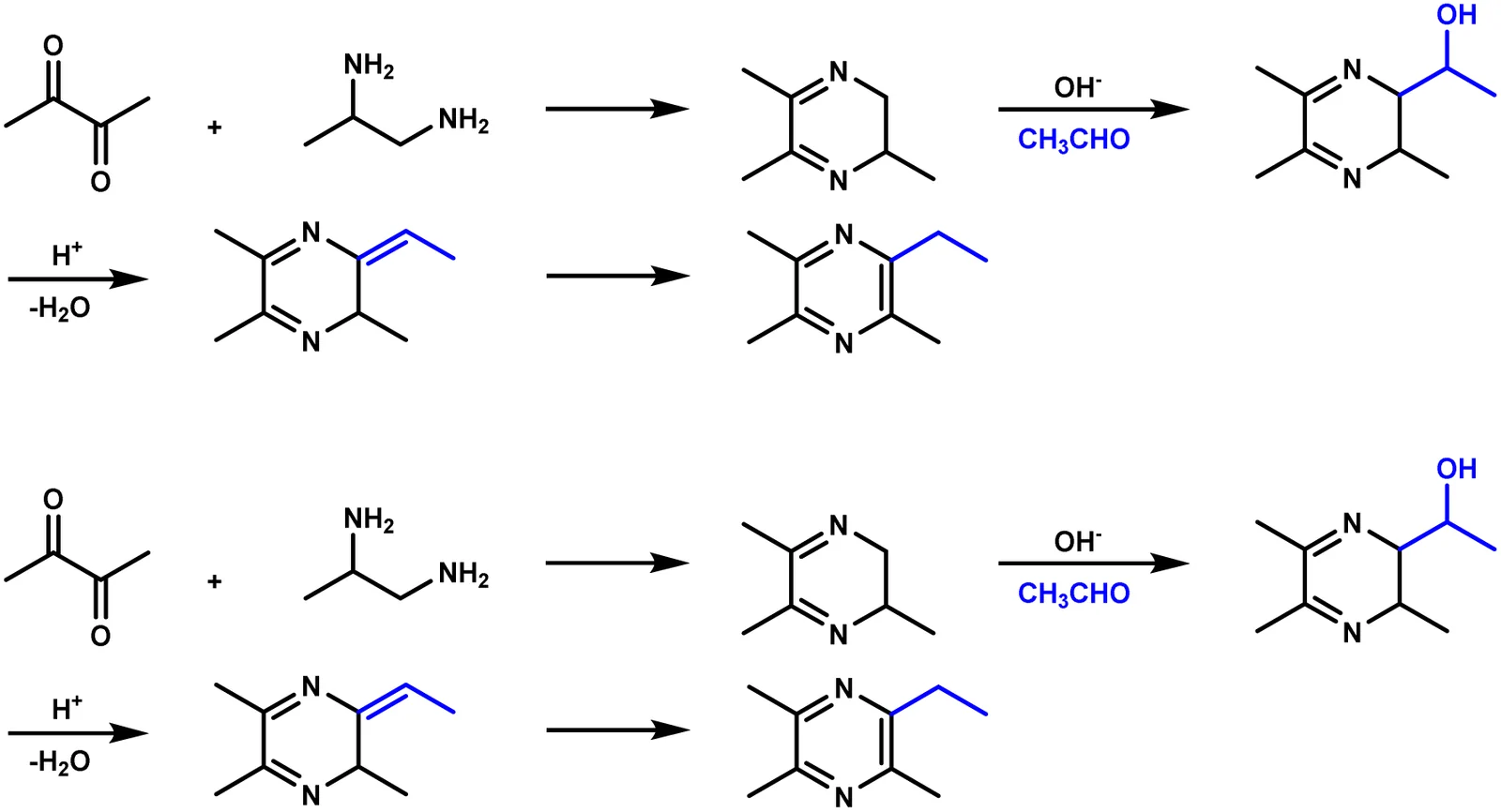
Formation mechanism of ethylpyrazine by-products.

A notable side reaction was identified when Akiyama and Shibamoto subjected 2,3,5-trimethyl-5,6-dihydropyrazine (obtained from the condensation of butane-2,3-dione with 1,2-propanediamine) to alkaline dehydrogenation in refluxing KOH/ethanol, followed by acid workup.^22^ Various ethylpyrazine by-products (*e.g.*, 2-ethyl-3,5,6-trimethylpyrazine) were formed; none of these were detectable before acid treatment. Mechanistic investigations suggested that under basic conditions, ethanol (the solvent) is slowly oxidised to acetaldehyde. Simultaneously, unreacted dihydropyrazine undergoes deprotonation at the C-6 methylene group to generate a carbanion, which attacks the carbonyl carbon of acetaldehyde to afford a hydroxyethyl addition intermediate (Scheme 4). This intermediate remains stable under basic conditions but, upon acid treatment, undergoes dehydration, cyclisation, and aromatisation to yield ethylpyrazines. Replacing ethanol with acetone gave isopropylpyrazines in 75% yield, confirming the generality of this solvent-mediated alkylation pathway.^23^ These observations highlight that acid treatment serves as the critical trigger for by-product formation; consequently, complete dehydrogenation and the avoidance of acid workup are essential when employing the Akiyama method to ensure pyrazine purity and stability.^24^

The α-diketone–diamine condensation–oxidation route provides reliable access to 2,3-disubstituted pyrazines, and high regioselectivity can be achieved with unsymmetrical diketones, as demonstrated by Flament.^18^ The MnO_2_-mediated variant developed by Akiyama operates under mild reflux conditions and yields stable, odourless products suitable for food applications.^19^ However, this approach typically requires two separate steps (condensation and oxidation) unless telescoped, and the use of stoichiometric MnO_2_ generates substantial solid waste (*ca.* 3 mol of MnO_2_ per mol of pyrazine), which contradicts the principles of atom economy. Furthermore, the solvent-mediated alkylation pathway can give rise to unwanted by-products if the reaction is not carefully controlled, and the method is incompatible with acid-sensitive substituents. Despite these drawbacks, the route remains a classic tool for alkylpyrazine preparation, particularly in flavour chemistry, although it has been increasingly superseded by greener catalytic alternatives in contemporary synthetic practice.

### 2.3 Dihydropyrazines: further reactions and fused derivatives

**Scheme 5:**
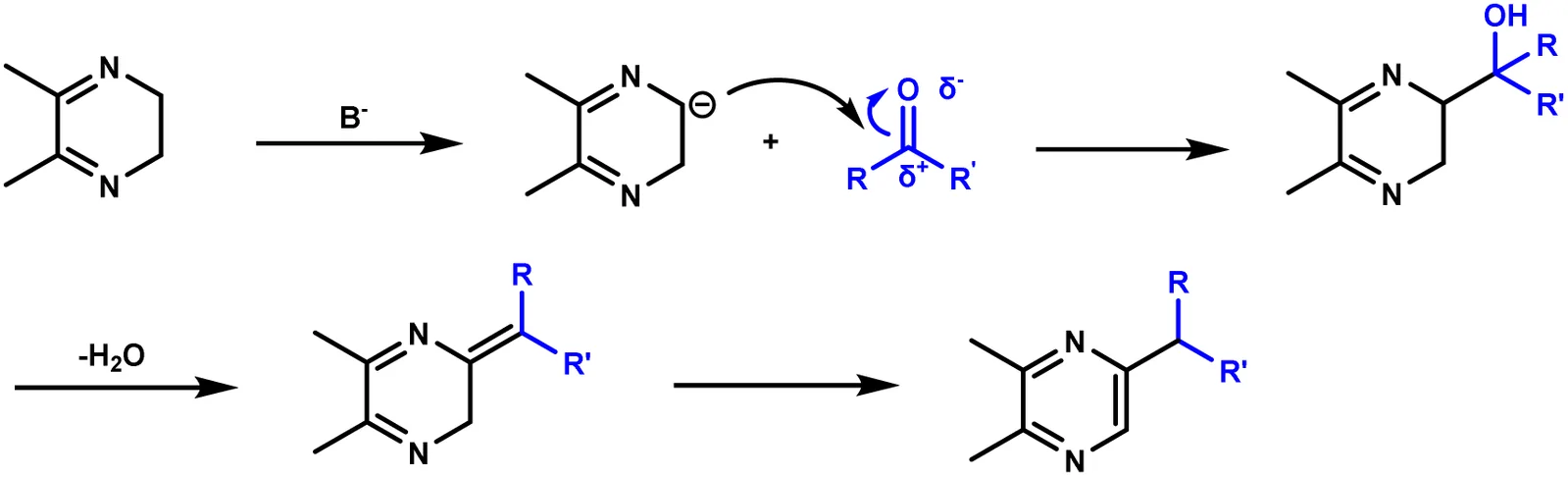
Condensation of dihydropyrazines with carbonyl compounds.

As a reactive intermediate, dihydropyrazines can not only be oxidatively dehydrogenated to pyrazines but also undergo condensation reactions with carbonyl compounds to directly construct trisubstituted pyrazines or fused pyrazine systems. In 1979, Flament and co-workers systematically investigated the reactions of 2,3-dihydropyrazines with various carbonyl compounds.^25^ In alkaline ethanol, 2,3-dihydropyrazines condensed smoothly with aliphatic, aromatic, terpenic and furanic aldehydes as well as ketones, affording trisubstituted pyrazines in high yields (Scheme 5). For example, the reaction of 5,6-dimethyl-2,3-dihydropyrazine with acetone gave 2,3-dimethyl-5-isopropylpyrazine in good yield. This method provided a convenient route for introducing sterically hindered or functionalized substituents onto the pyrazine ring.

**Scheme 6:**
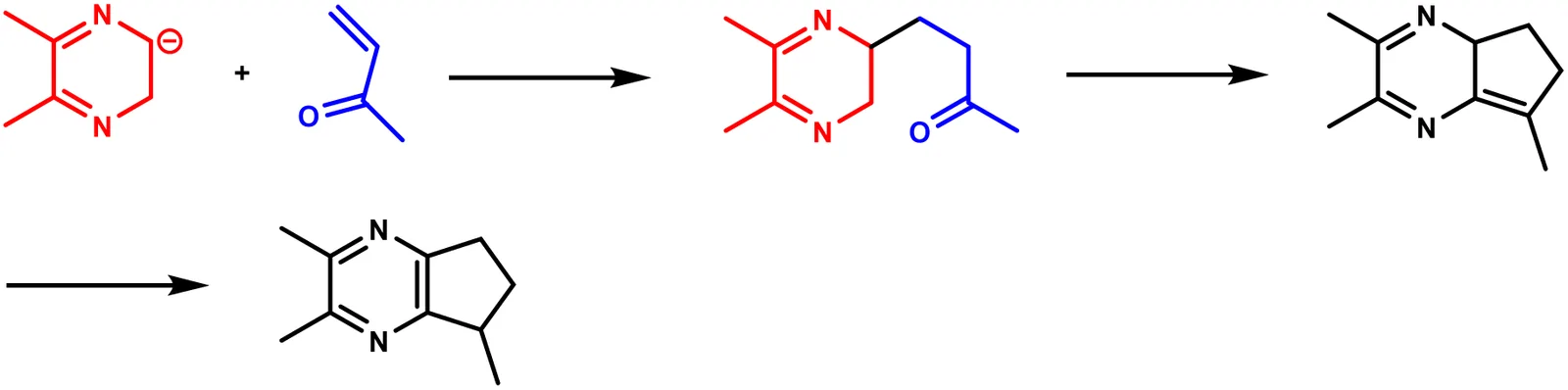
Synthesis of cyclopentapyrazines from dihydropyrazines.

When using α, β-unsaturated carbonyl compounds such as acrolein or methyl vinyl ketone, the reaction does not stop at simple condensation but proceeds further to cyclize, affording 6,7-dihydro-5*H*-cyclopenta[*b*]pyrazine (Scheme 6). This type of fused pyrazine possesses an intense roasted aroma and is an important flavor component in many baked foods. Prior to this work, Flament and co-workers had already synthesized analogous cyclopentapyrazines in 1976 *via* a multicomponent condensation of hydroxycyclopentenones with α-dicarbonyl compounds and ammonia, and identified them in the aroma of roasted beef.^26^ In contrast, the 1979 method reported by Flament is more straightforward, using readily available dihydropyrazines and α,β-unsaturated carbonyl compounds to construct the fused ring in one step *via* a Michael addition–aldol condensation cascade, thus avoiding the interference of by-products often encountered in multicomponent reactions.

These studies demonstrate that dihydropyrazines are not merely precursors to simple pyrazines, but can also be used to construct structurally more complex pyrazine derivatives through diverse condensation reactions, thereby providing greater flexibility for the synthesis of pyrazine compounds.

In 1990, Chiu and co-workers added pentanal or hexanal to a hydroxyacetone–ammonium acetate model system.^27^ The *in situ*-generated 2,5-dimethyl-3,6-dihydropyrazine intermediate underwent condensation with the long-chain aldehydes, successfully affording 2,5-dimethyl-3-pentylpyrazine, 2,6-dimethyl-3-pentylpyrazine and their hexyl analogues. In the absence of aldehyde, dihydropyrazine preferentially underwent dehydrogenation to 2,5-dimethylpyrazine; upon addition of aldehyde, the competing condensation pathway dominated, thereby introducing long-chain alkyl substituents. This work provided direct chemical evidence for the participation of aldehydes—arising from lipid oxidation—in the formation of long-chain alkylpyrazines *via* the Maillard reaction.

Collectively, these studies demonstrate that dihydropyrazines are not merely precursors to simple pyrazines, but can also be used to construct structurally more complex pyrazine derivatives—including trisubstituted pyrazines, fused bicyclic systems, and long-chain alkylpyrazines—through diverse condensation reactions, thereby providing greater flexibility for the synthesis of pyrazine compounds.

### 2.4 Further homogeneous condensation routes (1980–1990)

Beyond the classical routes and dihydropyrazine-based transformations, the 1980s witnessed the development of additional homogeneous condensation methods that expanded the synthetic scope of pyrazines. These methods, while not achieving industrial prominence, enriched the synthetic toolbox by enabling pyrazine formation from acyloins and pyrazinones under mild conditions.

#### 2.4.1 Reaction of acyloins with ammonium salts

**Scheme 7:**
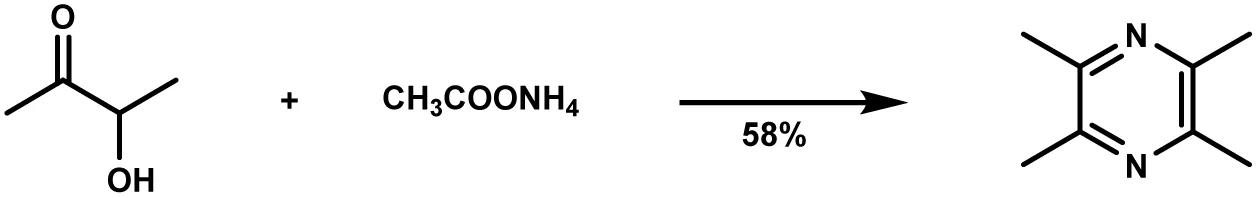
Synthesis of tetramethylpyrazine from acetoin (58%).

In 1988, Rizzi discovered that acyloins such as acetoin and hydroxyacetone react directly with ammonium salts under weakly acidic, mild conditions to afford alkylpyrazines.^28^ Using acetoin and ammonium acetate as starting materials at 120 °C for 15.5 h, tetramethylpyrazine was obtained in 58% yield (Scheme 7); even at room temperature, a yield of 13% was achieved. The reaction does not require oxygen, and mechanistic studies supported that it proceeds *via* an α-aminoketone intermediate rather than a diketone. This method provided a mild and efficient route to alkylpyrazines from acyloins, which are readily accessible from biomass-derived carbohydrates.

#### 2.4.2 Methylation of pyrazinones

**Scheme 8:**
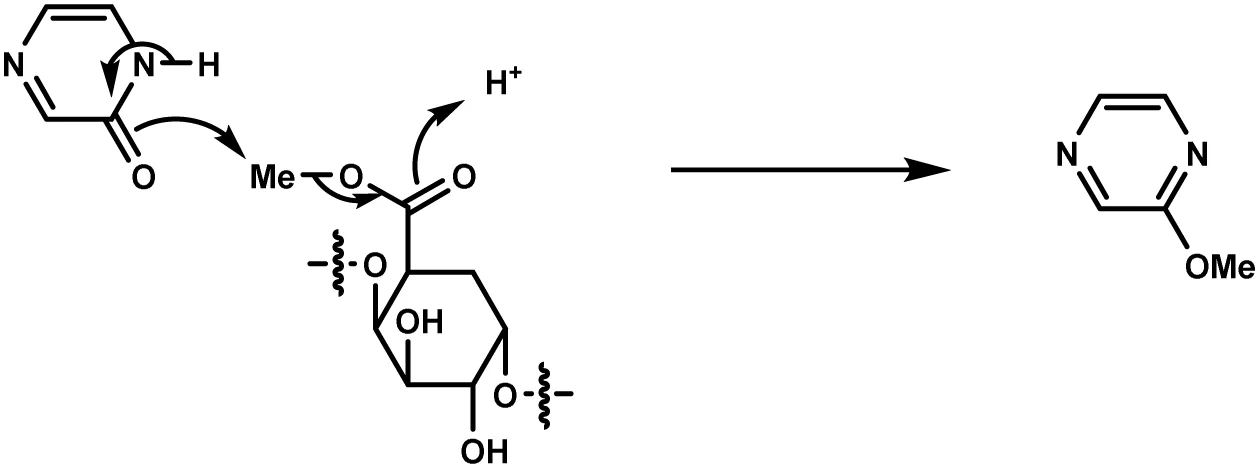
Formation of methoxypyrazine (0.09–0.62%).

Also in 1990, Rizzi exploited naturally occurring methylating agents present in foods—such as methyl esters of pectin, betaine, trigonelline, choline and lecithin—in reactions with 2(1*H*)-pyrazinone at 180 °C to achieve the biomimetic synthesis of methoxypyrazines.^29^ Although the yields were low (0.09–0.62%), this reaction demonstrated for the first time the feasibility of non-enzymatic methoxypyrazine formation. The mechanism involves nucleophilic attack of the pyrazinone oxygen atom on the methyl group, with the methyl esters of pectin being activated by the inductive effect of adjacent oxygen atoms and acid catalysis from uronic acid units (Scheme 8).

The homogeneous routes described in this section offered advantages that complemented the classical methods. The acyloin–ammonium salt approach operated under mild conditions and enabled the preparation of alkylpyrazines from readily available acyloin substrates.^28^ The methylation of pyrazinones, albeit low-yielding, demonstrated a direct pathway to methoxypyrazines that obviates the need for pre-functionalised halopyrazines.^29^ However, these methods generally suffered from poor yields (particularly the methylation reaction), restricted substrate scope, and limited applicability to diverse pyrazine targets. Consequently, they remain of interest principally as specialised synthetic tools rather than as general methods for pyrazine synthesis.

### 2.5 Summary

By 1990, the classical condensation–oxidation routes had been firmly established and further extended through a variety of homogeneous and biomimetic variants. Building on the contributions of Gabriel, Tutin, Cornforth and Rizzi, the α-aminoketone self-condensation was broadened from aryl to alkyl systems and the mechanisms of side reactions were elucidated. The α-diketone–diamine condensation–oxidation, refined by Ishiguro, Flament and Akiyama, enabled alkylpyrazines to be prepared under mild conditions with high efficiency and purity, meeting food-grade standards. Further condensation and cyclisation of dihydropyrazine intermediates demonstrated the potential to construct trisubstituted pyrazines, fused bicyclic systems and long-chain alkylpyrazines through reactions with aldehydes and α,β-unsaturated carbonyl compounds. Additional homogeneous methods introduced in the 1980s—acyloin–ammonium salt reactions and pyrazinone methylations—offered alternative synthetic entries to pyrazines, although their utility remained limited by low yields and narrow substrate scope.

Collectively, these foundational studies established the core reaction patterns and provided theoretical guidance for subsequent methodological advances. Nevertheless, all classical and homogeneous routes shared a fundamental limitation: they were primarily suited to simple alkyl- and aryl-substituted pyrazines and afforded minimal opportunities for late-stage functionalisation. At the same time, the demand for large-scale production of alkylpyrazines continued to drive the development of industrial heterogeneous catalytic processes, which are discussed in Section 3. In a parallel trajectory, the need for precise and diverse functionalisation of the pyrazine ring in pharmaceutical and materials science set the stage for the palladium-catalysed cross-coupling strategies presented in Section 4.

## 3. Industrial heterogeneous catalysis

While classical condensation–oxidation routes were predominantly practised in solution, the 1980s witnessed the emergence of industrially viable heterogeneous catalytic processes that enabled the gas-phase conversion of commodity feedstocks into alkylpyrazines with substantially improved yields and selectivities. This section covers three major catalytic systems—copper-chromite, zinc-chromite, and platinum-based catalysts—that laid the technological foundation for large-scale pyrazine production. Unlike the classical methods described in Section 2, these heterogeneous routes operate at elevated temperatures (300–450 °C) in fixed-bed reactors and convert readily available starting materials such as diamines, diols, and piperazine directly to pyrazines, thereby offering superior atom economy and process intensification. The mechanistic understanding gained from these systems also provided valuable insights into dehydrogenation and cyclisation pathways that later informed the design of milder homogeneous catalysts. The developments of this period are well documented in authoritative monographs and critical reviews.^7^

### 3.1 Copper–chromite catalysts

**Scheme 9:**
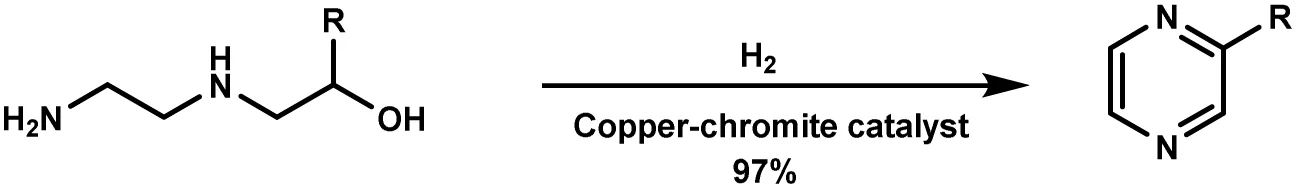
Cu–Cr-catalysed vapor-phase dehydrocyclization (>97% conversion).

Copper–chromite catalysts constituted an important catalytic system for vapour-phase pyrazine synthesis during this period. In 1988, Lee reported an improved catalyst reduction–activation procedure: a programmed temperature ramp from room temperature to 300–450 °C combined with a gradual increase in H_2_ concentration from 2% to pure hydrogen, which afforded a highly active Cu(0) phase.^30^ Vapour-phase dehydrocyclisation of *N*-(2-alkylhydroxy)ethylenediamine over this catalyst furnished pyrazine, 2-methylpyrazine and 2-ethylpyrazine, with conversions exceeding 97% and selectivities greater than 90%. Notably, the carrier gas must contain H_2_; otherwise, the catalyst undergoes rapid deactivation (Scheme 9).

### 3.2 Zinc–chromite catalysts

**Scheme 10:**
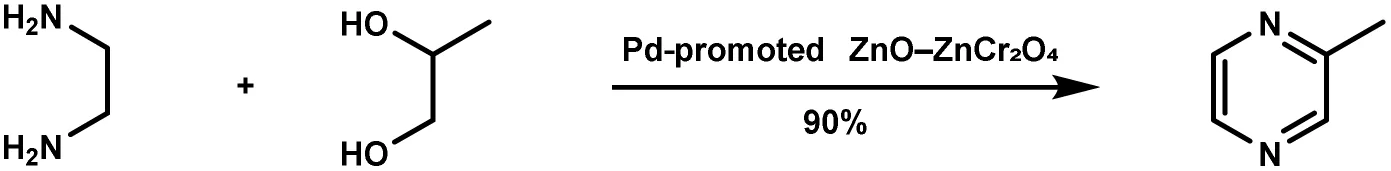
Synthesis of 2-methylpyrazine from ethylenediamine and propylene glycol.

In parallel with copper-based systems, Forni and co-workers developed a Zn–Cr–O catalyst (Zn/Cr = 3 : 1) doped with a small amount of palladium (0.5 wt%) as a dehydrogenation promoter for the one-step synthesis of 2-methylpyrazine from ethylenediamine and propylene glycol.^31^ Systematic optimisation of the catalyst preparation parameters—including the NaHCO_3_ concentration during co-precipitation and the reduction temperature—established the optimal reaction conditions: 370–400 °C and an ethylenediamine/propylene glycol molar ratio of 0.7, under which an ethylenediamine conversion of approximately 80% and a 2-methylpyrazine selectivity of approximately 90% were attained. This cyclisation provides a direct route to a key intermediate for the synthesis of pyrazinamide (2-amidopyrazine), a widely used antitubercular agent (Scheme 10).

**Scheme 11:**
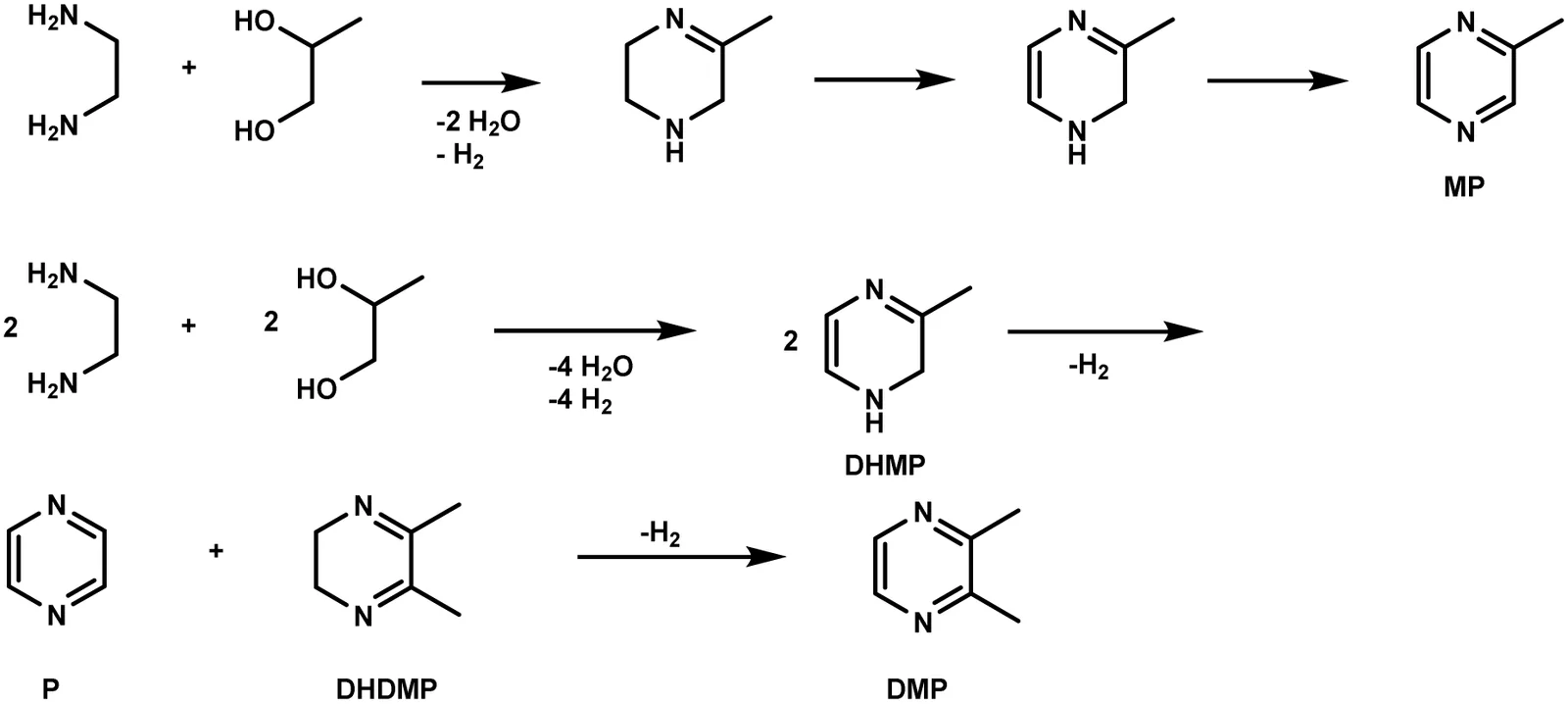
Pathways to MP and DMP over Pd–Zn–Cr–O.

Building on these findings, Forni and Miglio (1991) conducted an in-depth mechanistic investigation of this catalytic system. Detailed GC-MS analysis of the reactor effluent identified over 30 products and intermediates, revealing the multifunctional character of the catalyst.^32^ The results indicate that the target product is formed *via* a Pd-mediated hydrogenation–dehydrogenation sequence, in which the hydrogenation step relies on hydrogen transfer from propylene glycol rather than external H_2_. The pathway proceeds from ED and PG through MPIP and DHMP to MP. Simultaneously, the catalyst displays several additional catalytic functions, including dehydration, aldol condensation, double-bond isomerisation and steam dealkylation, which give rise to a variety of by-products. This study further clarified the origins of the major by-products: pyrazine (P) is formed predominantly by steam dealkylation of MP, whereas dimethylpyrazine (DMP) arises from transalkylation between adsorbed DHMP intermediates, rather than from direct methyl disproportionation of MP (Scheme 11).

**Scheme 12:**
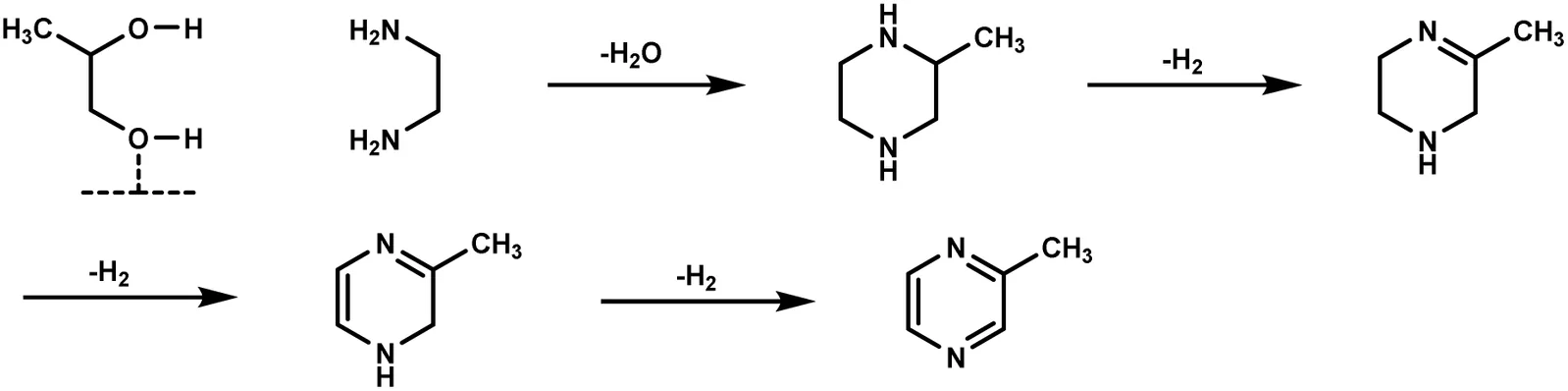
Formation of 2-methylpyrazine over Zn–Cr catalyst.

Based on TPD-TPR-MS analyses, Forni and co-workers proposed that, on the Zn–Cr catalyst surface, adsorbed propylene glycol and gaseous ethylenediamine first combine to form a methylpiperazine intermediate, which then undergoes dehydrogenation and aromatisation to afford methylpyrazine, rather than proceeding *via* an oxirane pathway (Scheme 12).^31^ This mechanistic picture was later consolidated in the comprehensive review by Higasio and Shoji (2001), which systematically summarised the catalytic systems developed from the late 20th century to the early 2000s and noted that Cu–Cr_2_O_3_, Cu–Zn–Cr, Zn–phosphate–Mn, Pd–MgCl_2_–Al_2_O_3_, and Ag-based catalysts are all effective for the conversion of ethylenediamine and propylene glycol into 2-methylpyrazine.^31^ Among these, copper–zinc catalysts also enabled the dehydrogenative aromatisation of piperazines to the corresponding pyrazines in good yields.

These investigations highlight that the defining characteristic of this catalytic system—its multifunctionality—is both an advantage and a challenge. On the one hand, the synergistic interplay of multiple catalytic functions enables one-step synthesis; on the other hand, the intricate by-product network compromises selectivity. This understanding suggests two directions for process improvement: first, tailoring the catalyst to selectively enhance the dehydrogenative cyclisation function while suppressing acid-catalysed side reactions; second, fine-tuning the process conditions (*e.g.*, feed ratio and steam level) to suppress the formation of specific by-products.

### 3.3 Platinum-based catalysts

A fundamentally different approach was explored by Isagulyants and co-workers (1990), who investigated the direct dehydrogenation of piperazine to pyrazine over Pt/Al_2_O_3_ catalysts.^33^ Over unmodified Pt/Al_2_O_3_, complete conversion of piperazine afforded pyrazine in only 43% yield, accompanied by 32% coke and 25% alkylpyrazines—a clear indication that the acidic support promoted undesired cracking and alkylation pathways. Introducing K_2_O to neutralise the acidic sites raised the yield to 59%. Subsequent modification with In_2_O_3_ (to enhance dehydrogenation) together with Re_2_O_7_ further increased the pyrazine yield to 80%, whilst reducing the alkylpyrazine and pyrrole contents to 5.1% and 0.6%, respectively.

This work demonstrated that the direct dehydrogenation of piperazine constitutes a viable route to pyrazine, and that by-products can be effectively suppressed through multi-component catalyst modification. More importantly, it illustrated a general principle of heterogeneous catalysis: the balance between the metal function (dehydrogenation) and the acidity of the support must be carefully tuned to avoid excessive reaction or coke deposition. Although more expensive than copper- or zinc-chromite catalysts, the Pt-based system offered the advantage of chromium-free operation and generated fewer high-boiling by-products.

### 3.4 Comparative assessment of industrial catalytic routes

The three heterogeneous catalytic systems developed between 1980 and 1990 each offered distinct advantages and limitations for industrial pyrazine synthesis. Copper-chromite catalysts delivered the highest single-pass conversions (>97%), but their activity depended on a strictly maintained reducing atmosphere, and rapid deactivation occurred in the absence of H_2_.^30^ Zinc-chromite catalysts achieved comparable selectivities (∼90%) and accepted a broader range of diamine–diol feedstocks, yet the mechanistic complexity arising from multiple competing pathways rendered precise control challenging.^31,32^ Platinum-based catalysts provided a chromium-free alternative for the dehydrogenation of piperazine, reaching 80% pyrazine yield after multicomponent promotion; nevertheless, the high cost of the precious metal and the need for sophisticated additive formulations hindered economic viability.^33^

All three systems operated at elevated temperatures (300–450 °C), entailing substantial energy input and placing demands on catalyst lifetime management owing to coking and sintering. Moreover, the presence of chromium in the copper- and zinc-chromite catalysts raised serious environmental and occupational health concerns, which subsequently motivated the search for chromium-free alternatives.

As shown in Table 2, the chromium-free alternatives offer distinct advantages over the historical chromium-containing systems. The ZnAl mixed oxide catalyst achieves comparable or superior selectivity (91.1–95.9%) while eliminating chromium-related toxicity. The Pt/CeO_2_/Al_2_O_3_ system operates at substantially lower temperature (180 °C *vs.* 340–440 °C) and converts biomass-derived diols directly. However, both chromium-free systems still rely on precious metals (Pt) or require high-temperature gas-phase operation, indicating that further catalyst improvements remain desirable.

**Table 2:** Comparison of historical chromium-containing catalysts and chromium-free alternatives for industrial pyrazine synthesis

Catalyst system	Reaction temperature	Key performance	Remarks	Ref.
**Chromium-containing catalysts (traditional)**				
Cu–Cr_2_O_3_	340–440 °C	Selectivity 98–100%	H_2_ carrier gas required; regenerable; Cr(vi) toxicity	8 and 30
Pd–Zn–Cr–O	∼387 °C (660 K)	Optimal at ∼660 K (mechanistic study)	TPD-TPR-MS mechanistic study; Cr(vi) toxicity	10

**Chromium-free alternatives (modern)**				
ZnAl mixed oxide	Gas phase	Selectivity 91.1–95.9%	Quasi-total conversion; thermally stable; Cr-free	12
Pt/CeO_2_/Al_2_O_3_	180 °C (0.7 MPa NH_3_)	Yield 94%	Biomass-derived diols; ≥6 cycles; Cr-free but contains Pt	13

It should be noted, however, that several industrially relevant parameters—including space-time yield, feed concentration, productivity, and detailed catalyst regeneration protocols—are not consistently reported in the primary literature cited for the chromium-containing systems. Most studies report selectivity and conversion under fixed operating conditions without providing long-term stability or continuous operation data. This data gap is itself a limitation of the available literature and highlights the need for future studies to report such parameters for more meaningful process comparisons.

Despite these drawbacks, the heterogeneous routes represented a decisive advance over the batchwise solution-phase condensations of the classical era. They established that pyrazines could be produced continuously from inexpensive commodity chemicals with high efficiency, thereby creating the technological foundation for the multi-thousand-tonne annual production capacity realised in later years. The principles of process intensification and catalyst multifunctionality revealed by these systems continue to guide contemporary heterogeneous catalysis research.

### 3.5 Summary

The decade from 1980 to 1990 witnessed significant progress in pyrazine synthesis through heterogeneous catalysis. Copper-chromite, zinc-chromite and platinum-based systems each enabled the efficient gas-phase conversion of commodity feedstocks—diamines, diols and piperazine—into alkylpyrazines with industrially attractive yields and selectivities. Detailed mechanistic investigations, especially on the Zn–Cr–O(Pd) system, uncovered the multifunctional character of these catalysts and identified key reaction intermediates, providing a rational basis for subsequent catalyst optimisation. Nevertheless, the intrinsic requirements of high-temperature operation, the sensitivity of catalyst activation, and the environmental liabilities associated with chromium limited the long-term sustainability of these routes. These constraints, coupled with the increasing demand for structurally diverse pyrazines in pharmaceutical discovery, catalysed the shift toward the palladium-catalysed cross-coupling strategies that are examined in Section 4, and later inspired the greener, biomass-derived methodologies discussed in Section 5.

## 4. From regioselective construction to precision functionalization

The 1990s marked a paradigm shift in pyrazine synthesis. While industrial heterogeneous catalysis continued to supply the commodity chemicals market (Section 3), the pharmaceutical industry's escalating demand for structurally diverse, multifunctionalised pyrazines—particularly kinase inhibitors and other bioactive agents—created an urgent need for methods capable of precise, late-stage ring functionalisation. This section traces how that need was met, beginning with early regioselective strategies based on electrocyclisation and cycloaddition (Section 4.1), which addressed the challenge of unsymmetrical pyrazine synthesis and set the stage for the transition-metal-catalysed approaches that followed. The subsequent adoption and maturation of palladium-catalysed cross-coupling reactions—including Suzuki, Stille, Sonogashira, Negishi, and Heck couplings, together with C–H activation and C–N/C–P bond-forming methods—provided a comprehensive toolbox for introducing aryl, heteroaryl, alkynyl, and alkyl groups under relatively mild conditions, and came to dominate pyrazine functionalisation by the early 2010s.

### 4.1 Regioselective *de novo* construction of pyrazines

The classical condensation–oxidation of α-diketones with α-diamines often yields mixtures of regioisomers when applied to unsymmetrical pyrazines, rendering purification difficult. In the early 1990s, two complementary non-palladium routes were reported that addressed this limitation, providing alternative strategies for constructing unsymmetrical pyrazines prior to the widespread adoption of cross-coupling methodology.

#### 4.1.1 Electrocyclisation strategy

**Scheme 13:**
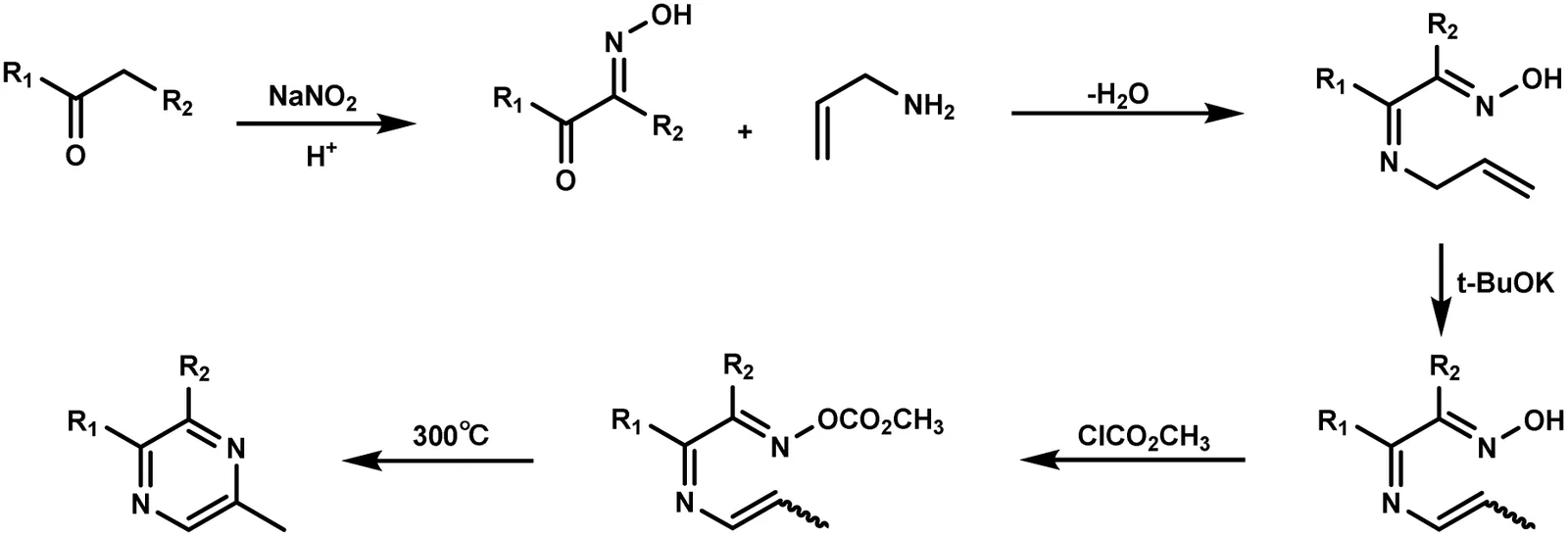
Synthesis of alkylpyrazines by electrocyclization strategy (61–69% yield).

In 1991, Büchi and Galindo devised a regioselective synthesis based on the thermal electrocyclisation of 1-hydroxy-1,4-diazahexatrienes.^34^ Condensation of α-oximinoketones with allylamine afforded imines, which were isomerised with potassium *tert*-butoxide to give 1-hydroxy-1,4-diazahexatrienes as mixtures of *E* and *Z* isomers. After *O*-methoxycarbonylation, short-contact pyrolysis at approximately 300 °C triggered a 6π-electrocyclisation–aromatisation sequence, delivering single regioisomers of alkylpyrazines—such as 2-methyl-5-ethylpyrazine and 2,5-dimethyl-3-ethylpyrazine—in 61–69% yield. The regioselectivity originates from the preferential cyclisation of the thermodynamically favoured *E* isomer (Scheme 13).

#### 4.1.2 Oxadiazinone–enamine cycloaddition strategy

**Scheme 14:**
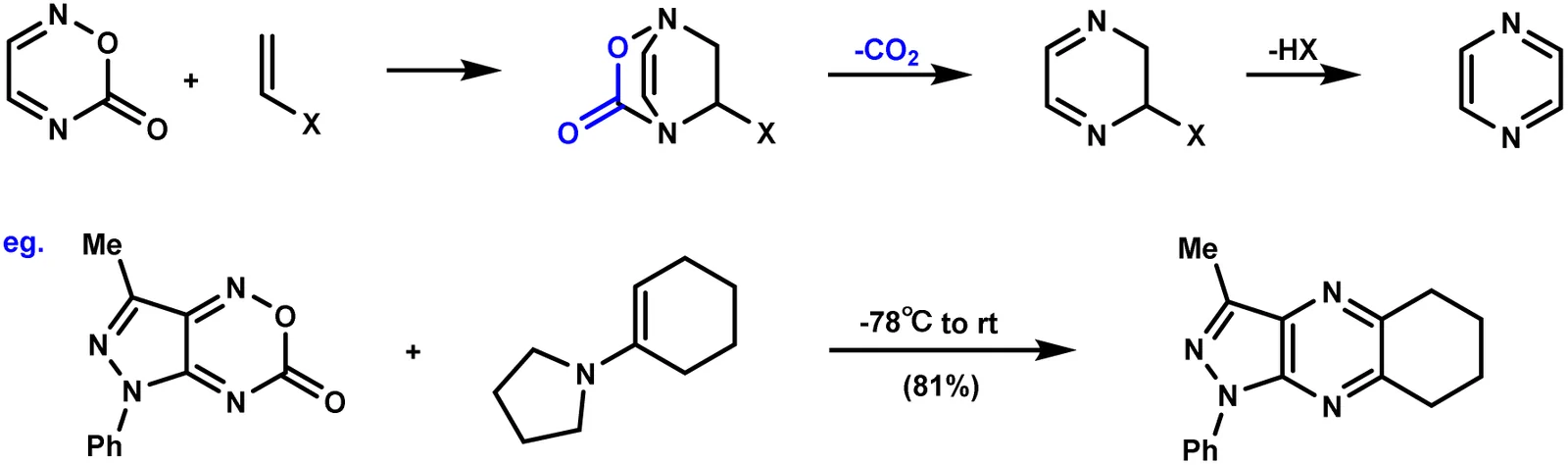
Oxadiazinone–enamine hetero-Diels–Alder cycloaddition.

In 1993, Ganesan and Heathcock reported a complementary regioselective route: cycloaddition of an oxadiazinone with electron-rich enamines, followed by loss of CO_2_ and HX, to aromatise directly to the pyrazine ring.^35^ For instance, reaction of the oxadiazinone with the pyrrolidine enamine of cyclohexanone proceeded instantaneously at room temperature, affording 5,6,7,8-tetrahydro-3-methyl-1-phenyl-1*H*-pyrazolo[3,4-*b*]quinoxaline in 81% yield. The reaction displayed high regioselectivity across a range of enamines and, notably, gave the first example of a cyclic *S*,*N*-ketene acetal that is stable in the absence of β-electron-withdrawing groups (Scheme 14).

The electrocyclisation and cycloaddition strategies reported by Büchi and Ganesan offered elegant solutions to the regioselectivity challenge in unsymmetrical pyrazine synthesis, delivering single regioisomers in good to excellent yields. Nevertheless, both methods required specialised substrates (α-oximinoketones or oxadiazinones), and Büchi's approach additionally relied on high-temperature pyrolysis. Coupled with the later emergence of more general palladium-catalysed cross-coupling methods, these constraints limited their uptake in medicinal chemistry. Nonetheless, they remain valuable as conceptually distinct approaches to regiocontrol and underscore the breadth of synthetic strategies available for pyrazine construction.

### 4.2 Palladium-catalysed cross-coupling for precision functionalization

The systematic application of palladium catalysis to pyrazine chemistry commenced in the early 1990s. It was discovered that chloropyrazines, particularly those bearing electron-withdrawing substituents, undergo efficient Pd(0)-catalysed coupling with a range of nucleophiles, thus providing a powerful tool for the late-stage functionalisation of the pyrazine ring.^8,9^ The broader development of cross-coupling methodologies and their applications in heterocyclic chemistry have been comprehensively reviewed.^36,37^

#### 4.2.1 Foundational studies: early explorations

The feasibility of palladium-catalysed cross-coupling on halopyrazines was first demonstrated in three independent studies between 1990 and 1997. These foundational investigations established the reactivity patterns and experimental conditions that would underpin all subsequent developments in pyrazine functionalization.

**Scheme 15:**
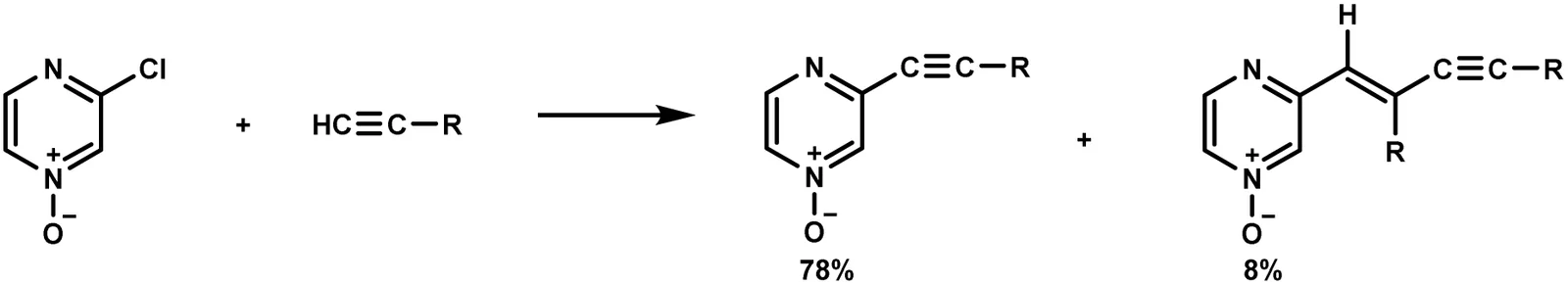
Palladium-catalysed coupling and codimerization of 3-chloropyrazine *N*-oxide with 1-hexyne (78% yield).

In 1990, Sato and co-workers reported the coupling of 3-chloropyrazine 1-oxide with 1-hexyne under Pd(PPh_3_)_4_/AcOK/DMF conditions, which afforded 3-(1-hexynyl)pyrazine 1-oxide in 78% yield, accompanied by an unexpected 8% of an alkenynylpyrazine formed *via* codimerisation of the alkynylpyrazine with a second molecule of the terminal alkyne.^38^ This observation revealed a potential tandem pathway in palladium-catalysed alkyne coupling and highlighted the diverse reactivity accessible from simple chloropyrazine precursors (Scheme 15).

**Scheme 16:**
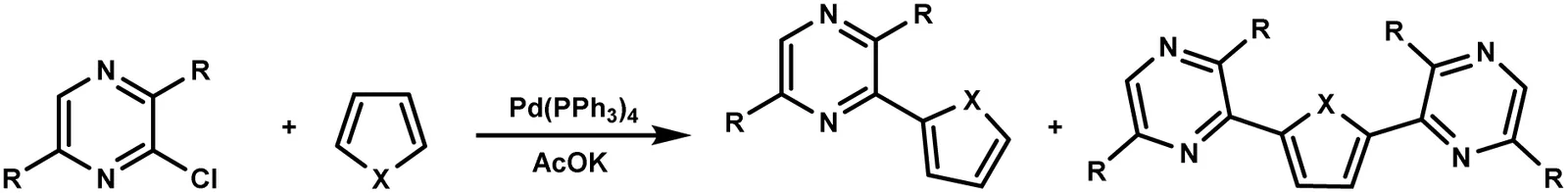
Pd-catalysed heteroarylation of 2-chloropyrazine (25–82% yield).

In 1992, Aoyagi, Ohta and co-workers systematically investigated the Pd(PPh_3_)_4_-catalysed cross-coupling of 2-chloro-3,6-dialkylpyrazines with a variety of aromatic heterocycles.^39^ In the presence of potassium acetate, the chloropyrazines coupled smoothly with furan, thiophene, pyrrole, benzofuran, benzothiophene, oxazole, thiazole, benzoxazole, benzothiazole and *N*-methylimidazole, affording the corresponding 2-heteroarylpyrazines in 25–82% yield. Remarkably, the reaction did not require prior conversion of the heterocycles into organometallic reagents and proceeded under mild conditions. Regioselectivity studies revealed that oxazole and thiazole reacted at the 5-position, whereas *N*-methylimidazole coupled at the 2-position (Scheme 16). This work established that direct C–H arylation could be achieved without pre-metallation, thereby significantly broadening the accessibility of heteroaryl-substituted pyrazines.^40–42^

**Scheme 17:**
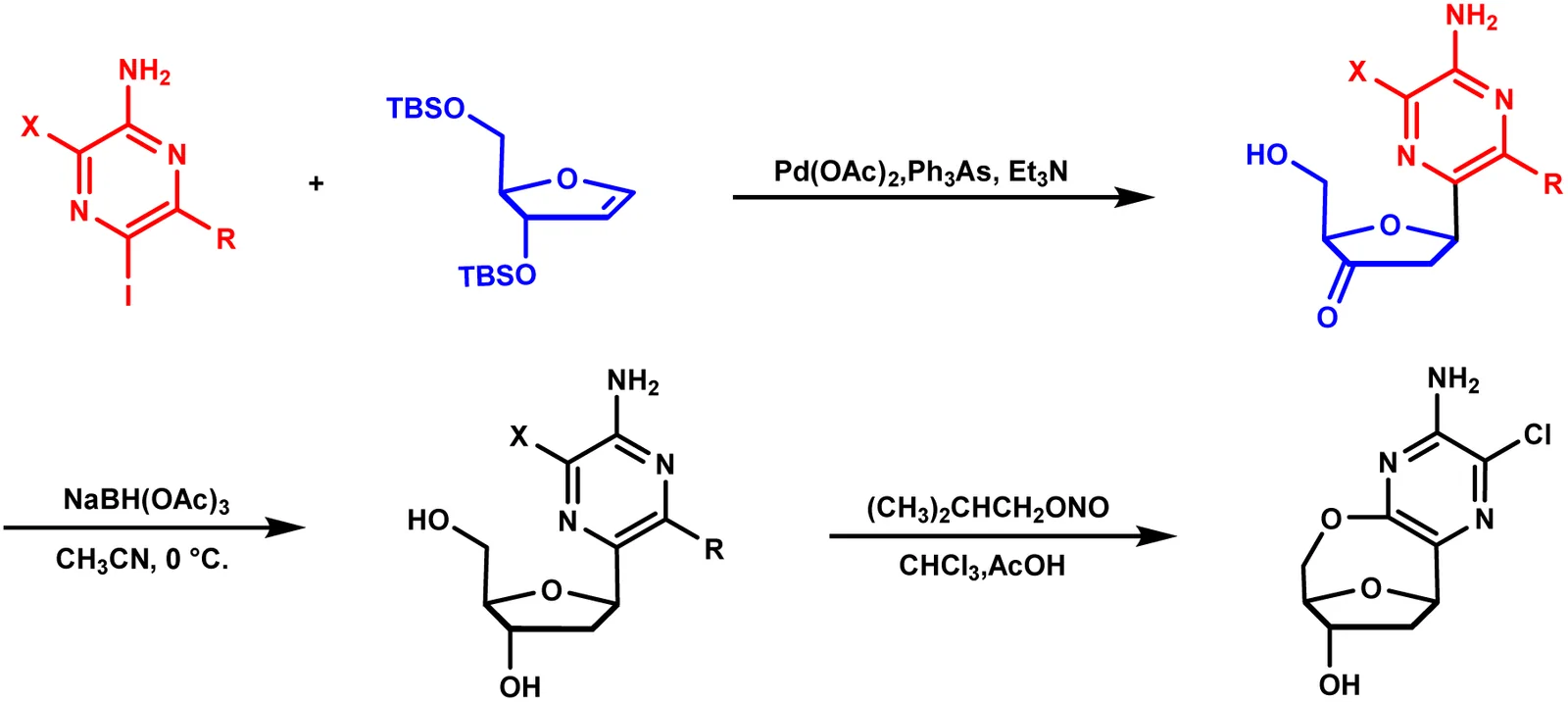
Pd-catalysed pyrazine–furanose glycal coupling (84% yield).

In 1997, Walker and co-workers extended palladium-catalysed cross-coupling to the synthesis of pyrazine C-nucleosides.^43^ Their group had previously reported the preparation and antiviral evaluation of pyrazinecarboxylic acid C-nucleosides.^44^ Building on this foundation, they employed 2-chloro-3,5-diamino-6-iodopyrazine or methyl 3-amino-6-iodopyrazine-2-carboxylate as substrates. Coupling with furanose glycals under Pd(OAc)_2_/Ph_3_As/Et_3_N conditions afforded 3′-keto intermediates, which after stereoselective reduction furnished 2-chloro-6-(2-deoxy-β-d-ribofuranosyl)-3,5-diaminopyrazine in 84% yield, together with related analogues. This work highlighted the unique advantages of palladium catalysis for constructing complex pyrazine C-nucleosides, a class of compounds with significant antiviral potential (Scheme 17). The contribution of Walker and Townsend's research to this area has been placed in a broader context by subsequent reviews.^45,46^

The early explorations of palladium-catalysed cross-coupling in the 1990s opened the door to pyrazine functionalisation on an unprecedented scale. The work of Sato, Ohta, and Walker collectively demonstrated that chloropyrazines are excellent substrates for Pd(0)-catalysed coupling, enabling the introduction of alkynyl, heteroaryl, and carbohydrate moieties under relatively mild conditions. Nevertheless, these early methods still required pre-functionalised starting materials (chloropyrazines) and, in many cases, relied on specific substitution patterns to achieve acceptable yields. The substrate scope, while impressive for its time, was limited in comparison with later advances. Despite these limitations, these foundational studies established the chemical principles and experimental protocols that would underpin the rapid expansion of pyrazine cross-coupling chemistry in the decades that followed.

#### 4.2.2 Suzuki–Negishi strategy for tetrasubstituted asymmetric pyrazines

A major advance in the precision functionalization of pyrazines came from Jeong and co-workers in 2010, who developed an efficient route from commercially available 2-chloro-3-methylpyrazine to tetrasubstituted unsymmetrical pyrazines and their *N*-oxides.^47^ The strategy involved a sequence of Suzuki coupling, regioselective *N*-oxidation, *N*-oxide-directed C–H arylation, chlorination, and Negishi coupling, enabling the sequential introduction of different aryl, alkyl, and heteroaryl groups onto the pyrazine ring with complete regiocontrol. This modular approach solved the long-standing problem of isomeric mixtures in unsymmetrical pyrazine synthesis (Section 2.2), providing a practical tool for structure–activity relationship studies in medicinal chemistry. The broader importance of C–H functionalization in this context has been highlighted in subsequent comprehensive reviews.^48^

The Jeong strategy represented a quantum leap in pyrazine synthetic capability. Prior to this work, the synthesis of tetrasubstituted pyrazines with four different substituents was exceedingly difficult, often requiring lengthy sequences with poor overall yields. The combination of Suzuki and Negishi couplings with regioselective *N*-oxidation provided a modular, programmable approach to pyrazine diversification. However, the method required multiple steps (typically five or more from the commercial starting material) and relied on precious palladium catalysts. Scalability for large-scale pharmaceutical production remained to be demonstrated, and the substrate scope, while broad, still required careful optimization for each new target. Despite these limitations, the Jeong strategy established a new benchmark for pyrazine functionalization and has been widely adopted in medicinal chemistry programs.

#### 4.2.3 Other cross-coupling modalities

Beyond Suzuki and Negishi reactions, a range of additional cross-coupling methods have been applied to pyrazine functionalisation. In 2013, Nikishkin and co-workers published a comprehensive perspective that systematically summarised progress in transition-metal-catalysed cross-coupling of pyrazine systems, covering diverse C–C and C–X bond-forming strategies from halopyrazine precursors.^8^ These encompassed Stille coupling (organotin reagents), Sonogashira coupling (terminal alkynes), Heck coupling (alkenes), Liebeskind–Srogl coupling (thioesters), as well as C–N and C–P bond formations. The breadth of available coupling methodology transformed the pyrazine ring from a relatively static scaffold into a versatile platform for generating structural diversity.

By 2010, the range of cross-coupling modalities available for pyrazine functionalisation was impressive. Each method offered distinct advantages: Sonogashira for alkynyl pyrazines, Heck for alkenyl derivatives, Suzuki for aryl pyrazines, and Buchwald–Hartwig for aminopyrazines. This toolbox enabled medicinal chemists to rapidly explore structure–activity relationships around pyrazine cores, thereby facilitating the development of numerous pyrazine-based drug candidates. However, several significant challenges persisted: the dependence on palladium catalysts, the requirement for pre-functionalised starting materials (halopyrazines), and the sensitivity of certain couplings to functional-group incompatibility. The cost of palladium, particularly for large-scale synthesis, further motivated the search for more sustainable alternatives—a theme that would dominate the next phase of pyrazine methodology development.^49^

### 4.3 Emerging post-2010 C–H functionalisation methods

Since 2010, the toolbox for pyrazine functionalisation has continued to expand beyond traditional palladium-catalysed cross-coupling, driven by the need to reduce reliance on pre-functionalised substrates. Direct C–H functionalisation bypasses pre-functionalisation. For pyrazine systems, palladium-catalysed C–H arylation has been demonstrated on scaffolds such as imidazopyrazine *via* a concerted metalation–deprotonation pathway, which achieves selective C_6_-arylation.^80^ Nickel catalysis has also enabled the direct C–H arylation of electron-deficient N-heteroarenes, including pyrazine, using organozinc reagents, with regioselectivity governed by the electrophilicity of the substrate.^81^ Silver-catalysed decarboxylative coupling has been developed for the regioselective acylation of pyrazine with α-oxocarboxylic acids.^82^ Although these emerging methods are not yet as broadly established as palladium-catalysed cross-coupling, collectively they illustrate the ongoing diversification of the pyrazine functionalisation landscape beyond halide-based coupling chemistry.

### 4.4 Summary

Between 1990 and 2015, palladium-catalysed cross-coupling reactions transformed pyrazine synthesis from a condensation-dominated field into a precision-oriented discipline. The early explorations of Sato, Ohta, and Walker established the fundamental reactivity of chloropyrazines in C–C bond formation, and the Jeong strategy demonstrated the power of sequential coupling reactions for constructing tetrasubstituted asymmetric pyrazines, addressing a long-standing challenge in pyrazine medicinal chemistry. The diverse array of coupling modalities—Suzuki, Negishi, Stille, Sonogashira, Heck, C–N, and C–P—furnished a comprehensive toolbox for pyrazine functionalisation.

Nevertheless, the reliance on precious palladium catalysts, the need for pre-functionalised halopyrazine substrates, and the environmental burden of many coupling protocols (organic solvents, phosphine ligands, elevated temperatures) increasingly drew scrutiny. These concerns, together with the broader movement toward green chemistry and sustainable synthesis, catalysed the development of the greener approaches that are discussed in Section 5.

## 5. Modern sustainable approaches

The 2010s saw the development of pyrazine synthesis methods that reduce or eliminate the use of organic solvents, transition metals, stoichiometric oxidants and harsh reaction conditions. Building on classical routes such as the condensation of α-diketones with 1,2-diamines, a variety of protocols have been introduced—including solvent-free, catalyst-free and air-oxidation procedures, metal-catalysed and electrochemical oxidative coupling, and novel cyclisation strategies, substantially expanding the accessible scope of pyrazines. This section surveys the major innovations from 2010 to 2026, covering solvent-free and catalyst-free protocols (Section 5.1), amino acid-based sustainable synthesis (Section 5.2), metal-catalysed and electrochemical oxidative coupling (Section 5.3), natural-product-derived and heterogeneous catalysis (Section 5.4), metal-free radical cyclisation (Section 5.5), other novel cyclisation strategies (Section 5.6), and emerging frontiers (Section 5.7). A comparative summary is presented in Section 5.8. The evolution of pyrazine chemistry towards more sustainable synthetic practices during this period has been well documented in several reviews (*e.g.*, metal-free multicomponent reactions and general green heterocyclic synthesis).^10,11,50,51^

### 5.1 Solvent-free and catalyst-free condensation

Although the classical condensation–oxidation routes are operationally well established, they typically require organic solvents, stoichiometric oxidants and extended reaction times. A series of improvements introduced in the 2010s significantly reduced the use of organic solvents, catalysts and stoichiometric oxidants in classical condensation–oxidation procedures.

**Scheme 18:**
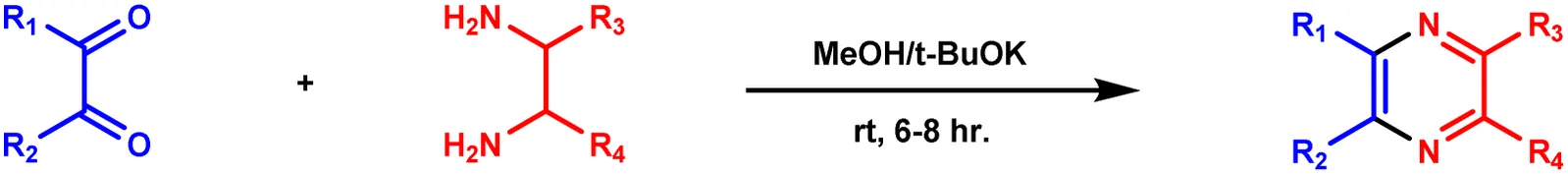
One-pot synthesis of 2,3-disubstituted pyrazines (76–88% yield).

In 2012, Ghosh and Mandal reported a room-temperature, one-pot procedure: in aqueous methanol containing a catalytic amount of potassium *tert*-butoxide as base, benzoins or 1,2-diketones reacted with ethylenediamine or diaminomaleonitrile for 6–8 h, during which the dihydropyrazine intermediate was spontaneously oxidised by air to afford the corresponding pyrazines in 76–88% yield.^52^ This method required neither external oxidants nor transition metals (Scheme 18), representing a significant improvement over the Akiyama MnO_2_ protocol.

Ghosh subsequently upgraded this method in 2017 to completely solvent-free conditions: ethylenediamine and 1,2-diketones (or α-hydroxyketones, α-bromoketones) were ground in a mortar for several minutes, then stirred at room temperature for 5–10 h. The products were isolated by simple filtration or column chromatography in 73–86% yield (1 mmol scale). This operation avoided any solvent or catalyst, offering significant advantages in terms of waste reduction and operational simplicity.^53^

In 2013, Huang and co-workers reported a catalyst-free synthesis of pyrazines from 1,2-diketones and aliphatic 1,2-diamines in polyethylene glycol (PEG) as the reaction medium.^54^ PEG could be recovered and reused, offering an alternative to volatile organic solvents in classical condensation reactions. However, the method was demonstrated primarily with aromatic 1,2-diamines for quinoxaline synthesis, and its application to aliphatic diamines for pyrazines was reported as a secondary scope.

**Scheme 19:**
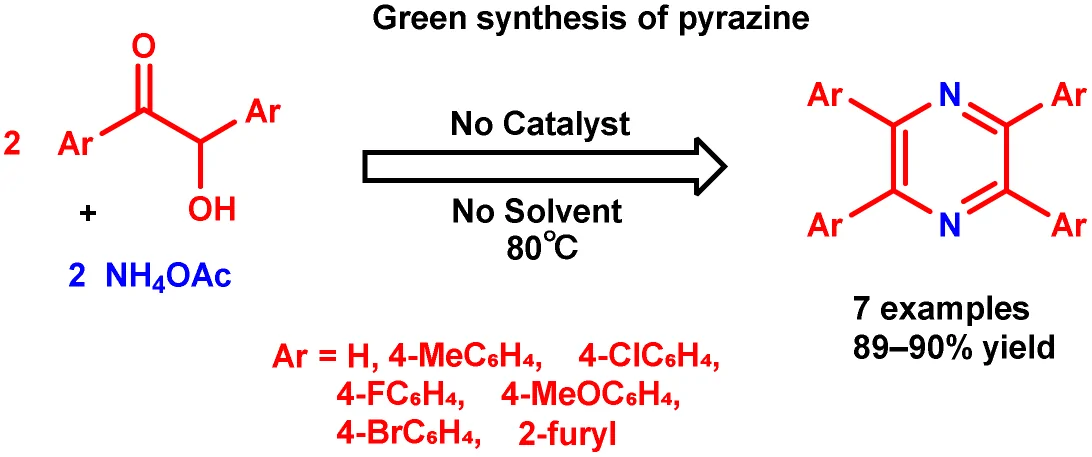
Solvent-free, catalyst-free one-pot synthesis of tetra-arylpyrazines.

In 2016, Tamaddon and co-workers developed a solvent-free, catalyst-free pseudo-four-component tandem reaction: benzoin derivatives were treated with ammonium acetate at 80 °C for 30–60 min, undergoing condensation, tautomerisation, self-condensation and air oxidation to afford tetraarylpyrazines in 89–93% yield.^55^ The method tolerated both electron-donating and electron-withdrawing substituents on the aryl ring and was also applicable to substrates such as phenylglyoxal and benzil (Scheme 19).

The solvent-free and catalyst-free protocols described above offer significant advantages in waste reduction and operational simplicity: they eliminate organic solvents and catalysts, use air as the terminal oxidant, and in some cases operate at room temperature. Huang's use of PEG as a recyclable solvent provides a practical alternative when a solvent phase is required. However, the substrate scope varies among the methods. For the preparation of simple alkyl- and arylpyrazines, these protocols offer operationally straightforward and low-waste alternatives to classical routes, though their applicability to complex or highly functionalized targets remains more constrained than palladium-catalysed cross-coupling (Section 4).

### 5.2 Amino acid-based sustainable synthesis

Amino acids, as renewable nitrogen sources derived from biomass, offering an alternative to petroleum-based feedstocks for pyrazine synthesis. Two complementary strategies emerged in the 2010s: direct reduction–cyclisation of amino acid esters and the Dakin–West-based approach.

**Scheme 20:**
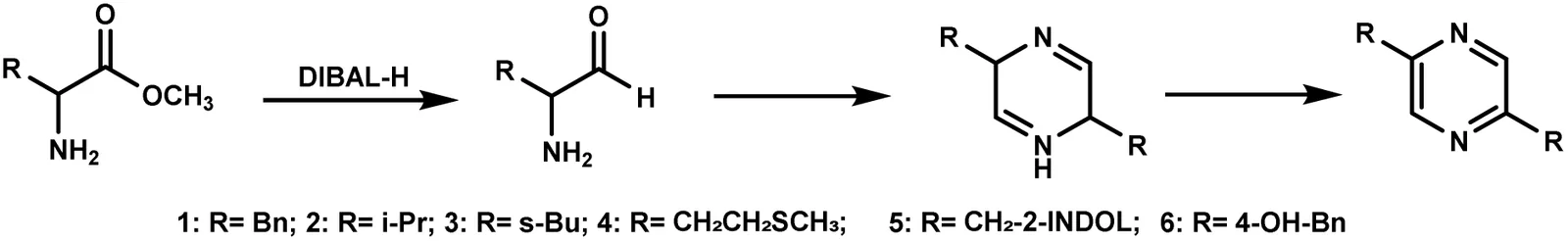
Symmetric 2,5-disubstituted pyrazines from α-amino esters *via* one-pot (∼50% overall yield).

In 2013, Rojas and co-workers reported a three-step, one-pot procedure: natural α-amino acid methyl esters were reduced with DIBAL-H at −78 °C to the corresponding α-amino aldehydes, which underwent spontaneous dimerisation–condensation followed by air oxidation to afford symmetric 2,5-disubstituted pyrazines in approximately 50% overall yield (*ca.* 80% per step) on a 0.6 mmol scale.^56^ The method was applicable to phenylalanine, valine, isoleucine, methionine, tryptophan and tyrosine, but delivered only symmetric products (Scheme 20). This restriction to symmetric pyrazines reflects the dimeric nature of the condensation, in which two identical α-amino aldehyde molecules condense to form the pyrazine ring.

**Scheme 21:**
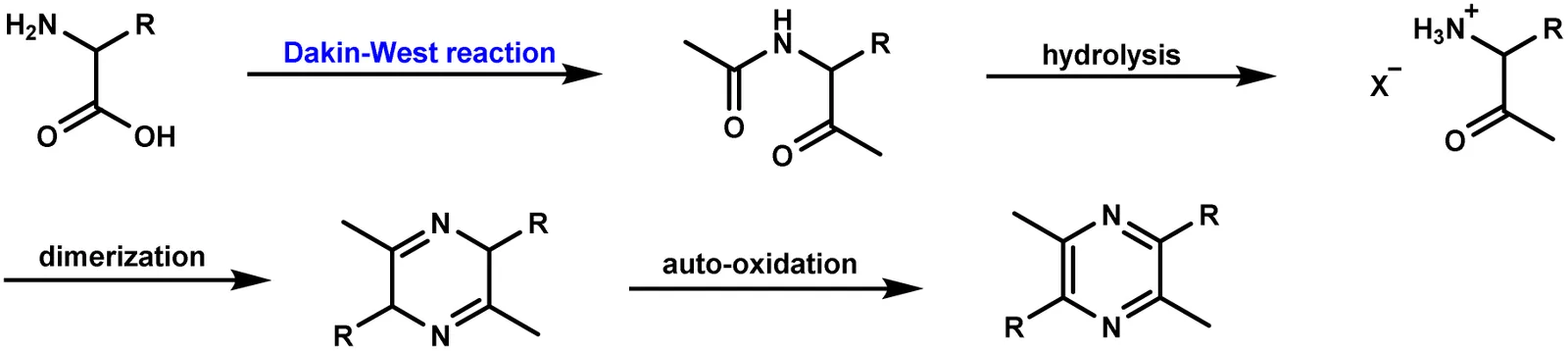
Synthesis of pyrazine from amino acids *via* the Dakin–West reaction (33–64% yield).

In 2020, Würdemann and co-workers revisited the classic Dakin–West reaction: amino acids were treated with acetic anhydride, triethylamine and DMAP to give α-acetamidoketones, which were then deacetylated with acid and cyclised using H_2_O_2_/TEA in methanol, furnishing dimethylpyrazines in three steps.^57^ With glutamic acid, lysine, methionine, histidine and tyrosine as substrates, overall yields ranged from 33 to 64%, and the tyrosine derivative was obtained in 63% yield over three steps. The process was amenable to scale-up to 100 g, demonstrating its practical feasibility on a preparative scale (Scheme 21). The scope and mechanism of the Dakin–West reaction have been comprehensively reviewed.^58^

The amino acid-based routes utilise renewable, biomass-derived feedstocks, which distinguishes them from petroleum-based approaches to pyrazine synthesis. However, a balanced assessment must also consider the limitations. The Rojas method is operationally straightforward but limited to symmetrical products and requires DIBAL-H at −78 °C, incurring substantial energy input. The Würdemann Dakin–West route offers broader functional group tolerance and has been demonstrated on a 100 g scale, but consumes stoichiometric acetic anhydride, triethylamine, DMAP, and hydrogen peroxide—reagents that carry their own environmental burdens in terms of synthesis and waste disposal. Thus, while the feedstocks are renewable, the overall process mass intensity, reagent consumption, and energy requirements remain significant considerations. Future developments are likely to concentrate on one-pot catalytic transformations that convert amino acids directly into pyrazines without the isolation of intermediates.

### 5.3 Metal-catalysed and electrochemical oxidative coupling

The direct construction of the pyrazine ring from simple ketones *via* C–H/N–H oxidative coupling, bypassing the need for pre-prepared α-diketones, emerged as an alternative strategy during this period. Two major approaches emerged: copper-catalysed aerobic oxidation and electrochemical oxidation.

**Scheme 22:**
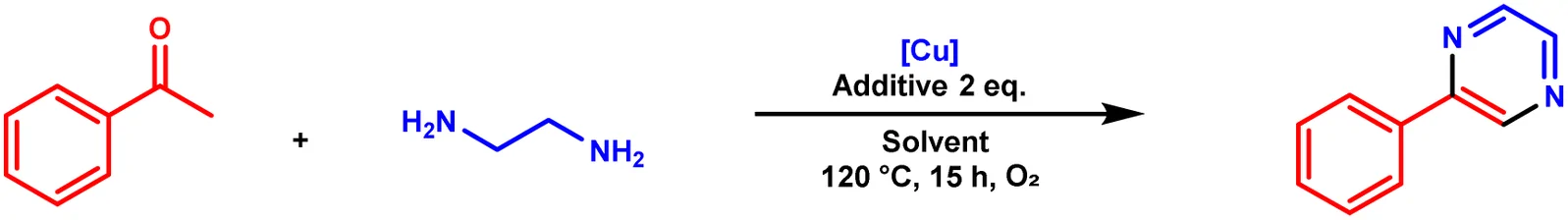
Copper-catalysed aerobic oxidative coupling to symmetric tetrasubstituted pyrazines (43–81% yield).

In 2015, Wu and co-workers reported a copper-catalysed aerobic oxidative system: employing 10 mol% CuI as catalyst and LiCl as additive in DMA/triethylamine (2 : 1, v/v) under 1 atm O_2_ at 120 °C for 15 h, aryl methyl ketones and cycloalkyl ketones (0.5 mmol) reacted with ethylenediamine (0.5 mmol) to afford symmetric monosubstituted pyrazines in 43–81% yield.^59^ Mechanistic studies proposed that the reaction proceeds *via* intramolecular cyclisation of an enamine cation radical; X-ray absorption fine structure (XAFS) analysis revealed that the active copper(ii) centre is coordinated by two nitrogen atoms (2.04 Å) and two oxygen atoms (1.98 Å). This work achieved the direct conversion of simple ketones into pyrazines, eliminating the need for pre-prepared α-diketones. The study proposed a radical-polar crossover mechanism and used XAFS characterisation to follow the evolution of the copper catalyst during the reaction (Scheme 22).

**Scheme 23:**
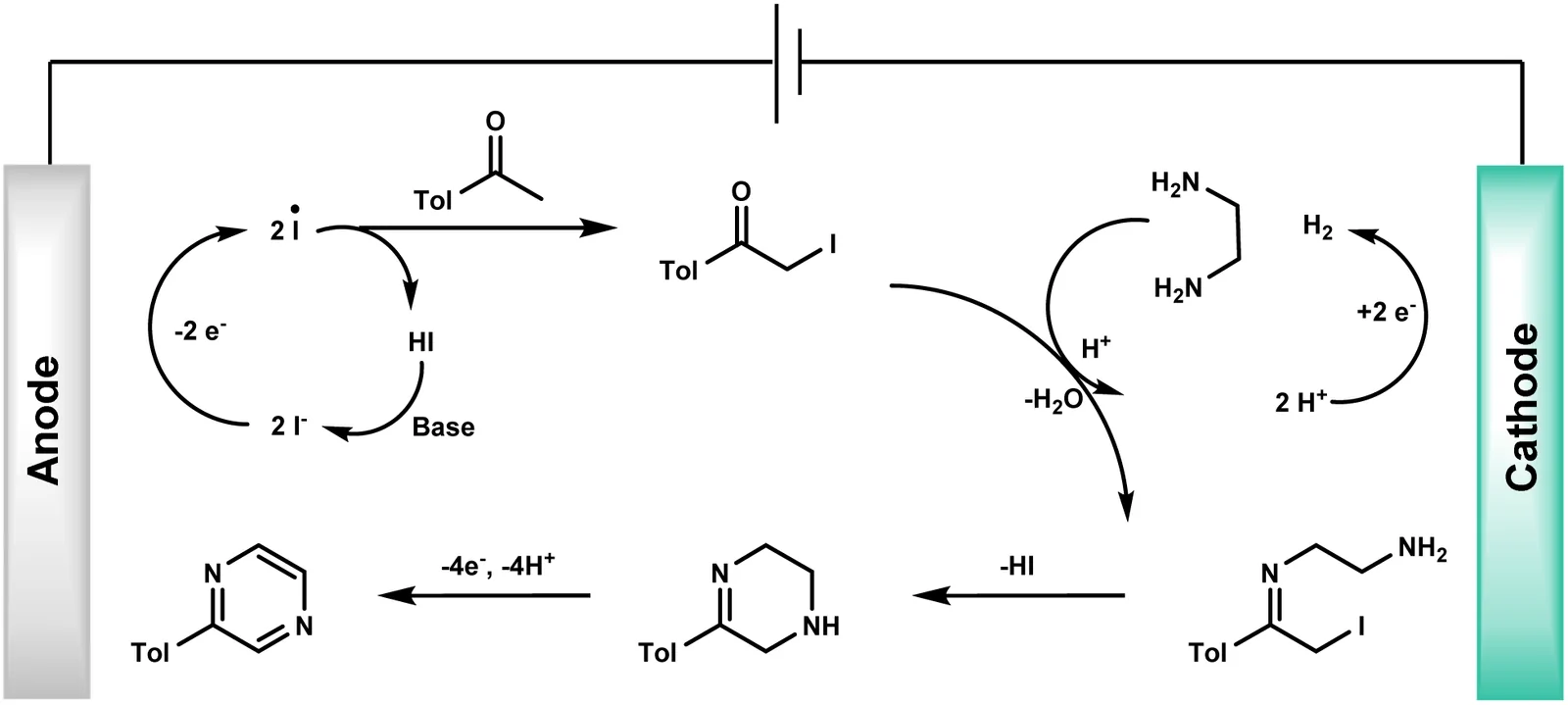
Electrochemical [4 + 2] annulation for pyrazines: mechanism.

In 2019, Liu and co-workers further developed an electrochemical variant: in an undivided cell equipped with a graphite rod anode and platinum plate cathode, with KI as redox mediator and TsOH·H_2_O as acid, aryl ketones underwent [4 + 2] dehydrogenative annulation with ethylenediamine under constant current electrolysis (13 mA) at 100 °C for 5.7 h to give the corresponding pyrazines.^60^ This protocol required neither transition metals nor chemical oxidants, accommodated substrates bearing halogens, fused rings and heterocycles, and was amenable to scale-up to 10 mmol. A proposed mechanism involves anodic oxidation of I^−^ to iodine radical, generation of α-iodoketone, and subsequent condensation with the diamine followed by cyclisation and dehydrogenative aromatisation (Scheme 23). Recent advances in electrochemical cascade cyclisation for heterocycle synthesis have been comprehensively reviewed.^61^

The copper-catalysed and electrochemical oxidative coupling methods enable the direct conversion of simple ketones into pyrazines without the need for pre-prepared α-diketones or α-aminoketones, offering a more direct route than classical condensation–oxidation sequences. The electrochemical variant avoids transition-metal catalysts and chemical oxidants, though it requires energy input, a platinum cathode, and redox mediators. Both methods are currently limited to symmetrical monosubstituted pyrazines (derived from aryl methyl ketones) and have not been extensively explored for unsymmetrical or highly substituted targets. Although the substrate scope continues to expand, it remains constrained in comparison with palladium-catalysed cross-coupling. Nonetheless, these strategies demonstrate the potential of oxidative coupling and electrosynthesis for streamlining pyrazine construction.

### 5.4 Natural product-derived and heterogeneous catalysis

The 2020s have witnessed the emergence of natural product-based catalysis and improved heterogeneous catalytic systems as alternatives to conventional industrial processes.

**Scheme 24:**
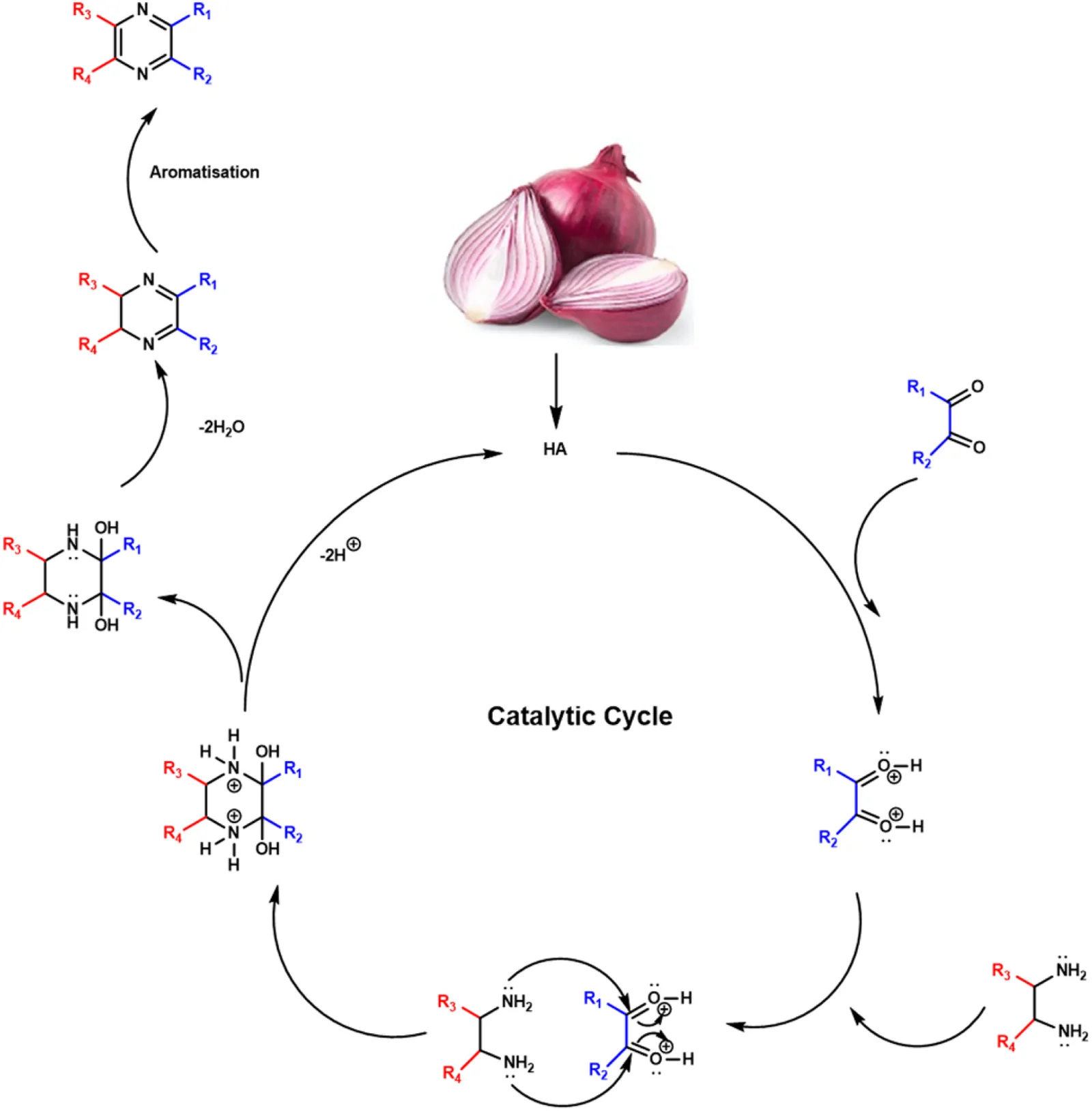
Onion extract-catalysed synthesis of pyrazine derivatives (85–96% yield).

In 2021, Dasgupta and co-workers reported an onion extract-catalysed synthesis of pyrazines.^62^ Onion extract, which is rich in organic acids such as caffeic acid, ferulic acid and tannic acid, catalysed the condensation of 1,2-diketones (1 mmol) with 1,2-diamines (1 mmol) in ethanol at room temperature; subsequent air oxidation afforded pyrazine derivatives in 85–96% yield. The catalyst could be recycled up to four times, and the procedure is both operationally simple and low-cost (Scheme 24). The mechanism is proposed based on the acidic composition of the extract; the individual steps remain speculative.

**Scheme 25:**
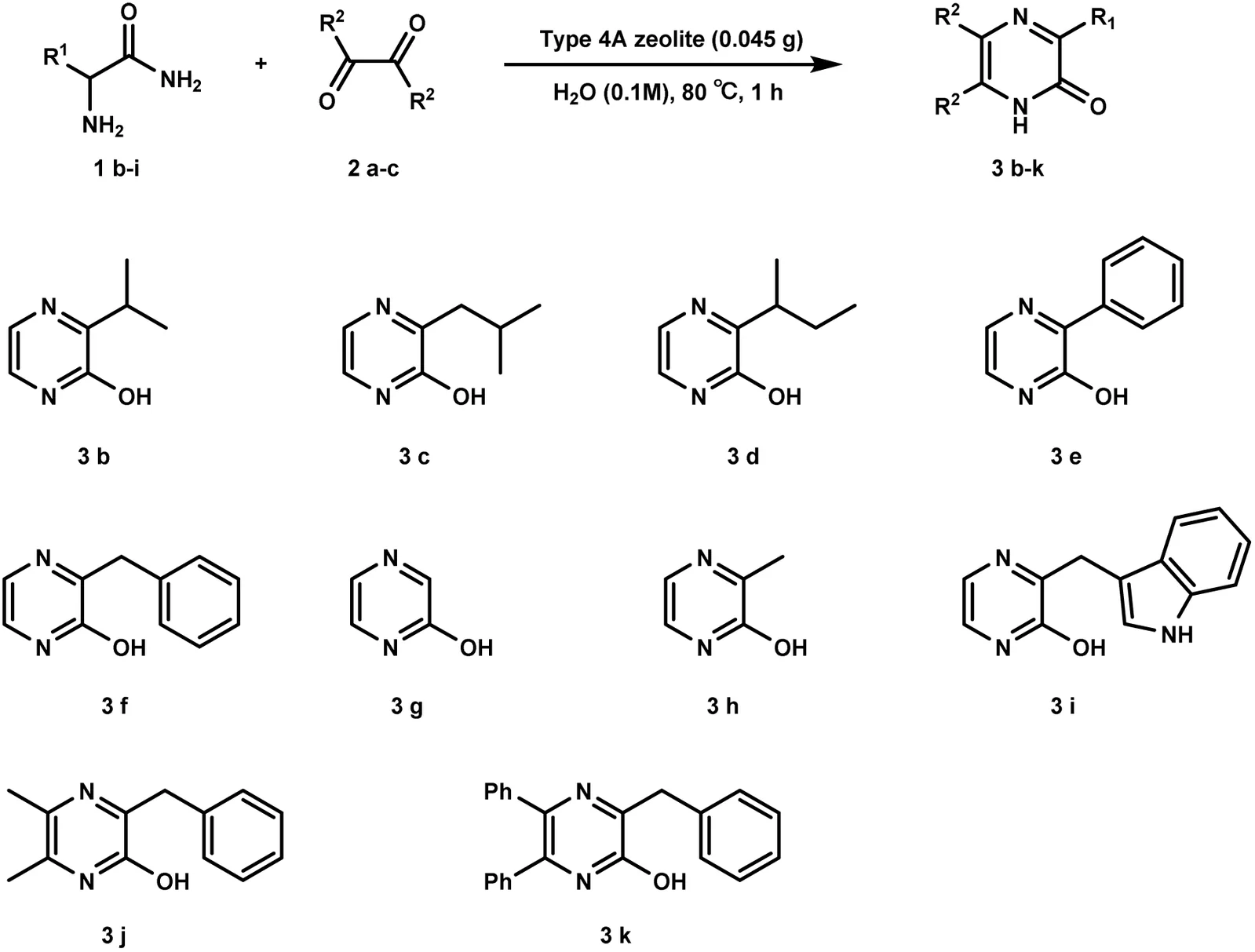
Zeolite-catalysed aqueous synthesis of pyrazinone derivatives.

Also in 2021, Hisada and co-workers employed Linde Type A zeolite as a heterogeneous catalyst in water for the condensation of amino acid amides with glyoxal to give pyrazinone derivatives in 49–94% yield.^63^ This system avoided organic solvents and corrosive reagents and was successfully translated into a continuous-flow process (Scheme 25). Recent reviews have highlighted the broad utility of heterogeneous catalysts in heterocyclic synthesis.^64^

**Scheme 26:**
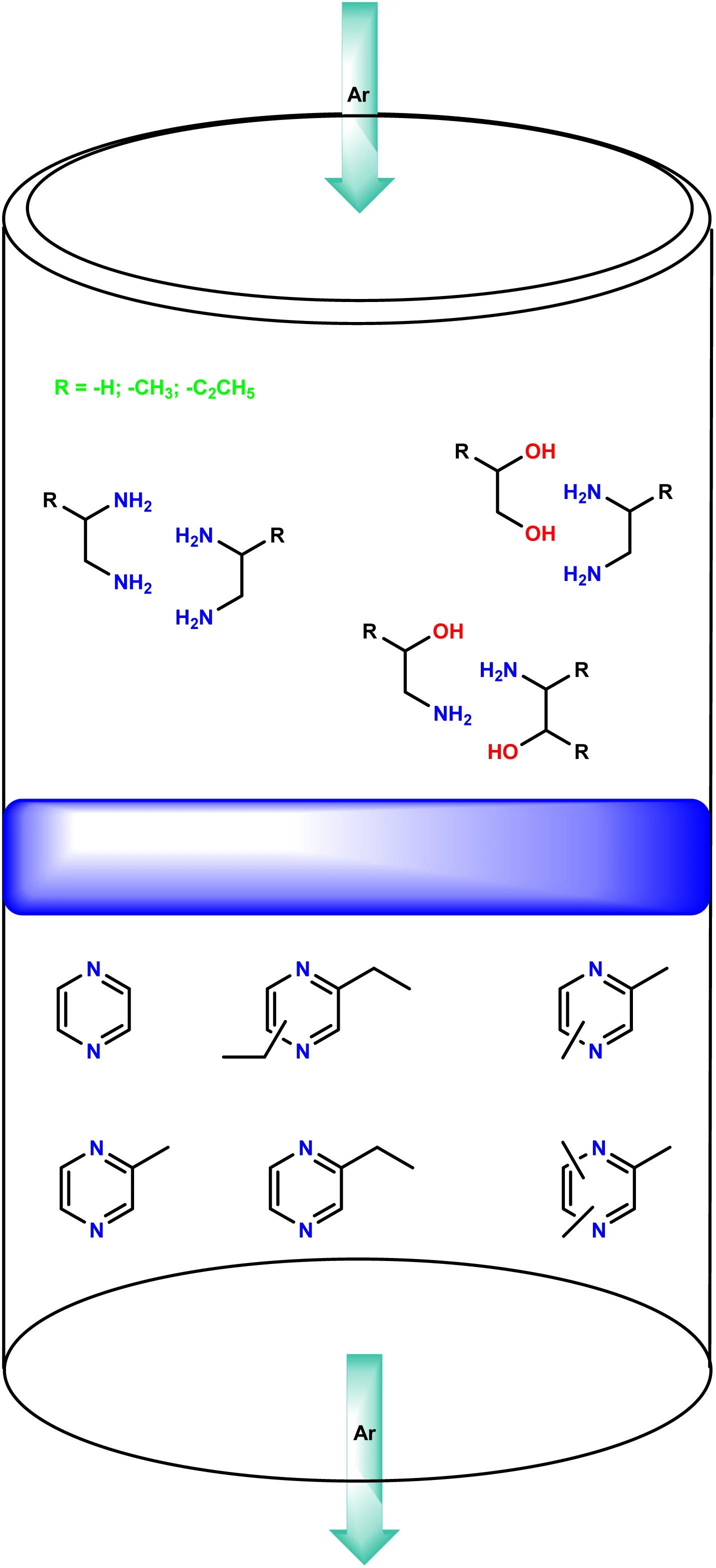
ZnAl mixed oxide-catalysed gas-phase synthesis of alkylpyrazines.

In the same year, Teodorescu and co-workers applied a ZnAl mixed oxide catalyst prepared by mechanochemical synthesis (*via* calcination of layered double hydroxides) in a gas-phase fixed-bed reactor for the dehydrocyclisation of diamines, diamine–diol mixtures and α-hydroxyamines.^65^ For instance, the self-reaction of 1,2-propanediamine gave dimethylpyrazine with 95.9% selectivity, whereas the condensation of ethylenediamine with 1,2-propanediol afforded 2-methylpyrazine with 91.1% selectivity (Scheme 26). The catalyst exhibited high thermal stability and reusability, and avoided the use of chromium.

**Scheme 27:**
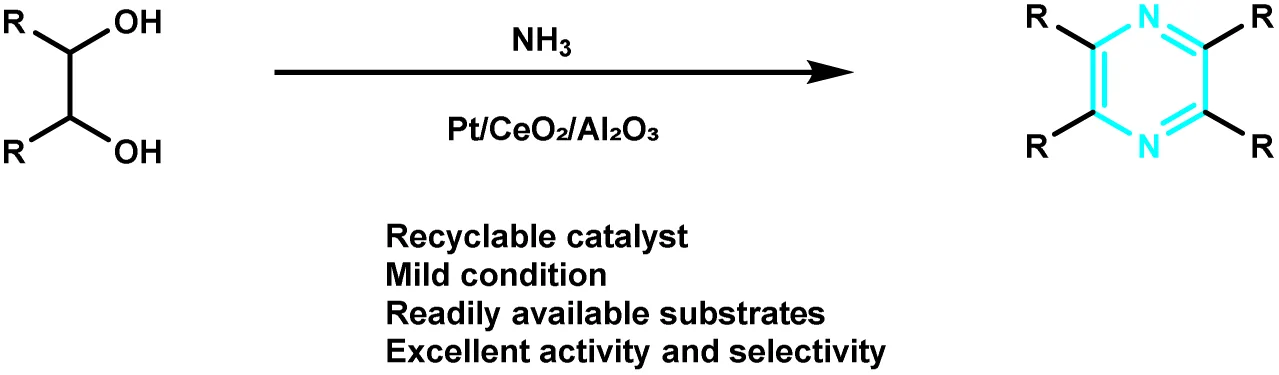
Pt/CeO_2_/Al_2_O_3_-catalysed amination of biomass-derived diols to pyrazines (44–77% yield).

In 2025, Zhou and co-workers further developed a Pt/CeO_2_/Al_2_O_3_ heterogeneous catalyst for the direct coupling of biomass-derived vicinal diols—such as 2,3-butanediol, ethylene glycol and 1,2-propanediol—with ammonia.^66^ Under 180 °C and 0.7 MPa NH_3_, 2,3-butanediol was converted to 2,3,5,6-tetramethylpyrazine in up to 94% yield. By optimising the CeO_2_ loading to an optimal value of 13 wt%, the Pt dispersion, electron density and acid/base properties of the catalyst were tuned, raising the pyrazine yield from 44% to 77% (Scheme 27). The catalyst could be reused six times without significant loss of activity.

The natural product-derived and heterogeneous catalytic systems developed in the 2020s offer alternatives to conventional industrial processes. The onion extract catalyst offers a simple and low-cost approach for laboratory-scale synthesis. However, as a crude biological extract, its composition can vary with source, cultivar, season, and extraction method, and its mechanistic basis and long-term stability have not been systematically investigated. These limitations should be considered when evaluating the method's potential for scalable applications. The zeolite and ZnAl mixed oxide systems are chromium-free, distinguishing them from the earlier copper-chromite and zinc-chromite catalysts. The Pt/CeO_2_/Al_2_O_3_ system developed by Zhou converts biomass-derived diols into pyrazines with catalyst recyclability, though it relies on a precious metal. The high temperatures required for gas-phase processes also represent an energy consideration.

### 5.5 Metal-free radical cyclisation

**Scheme 28:**
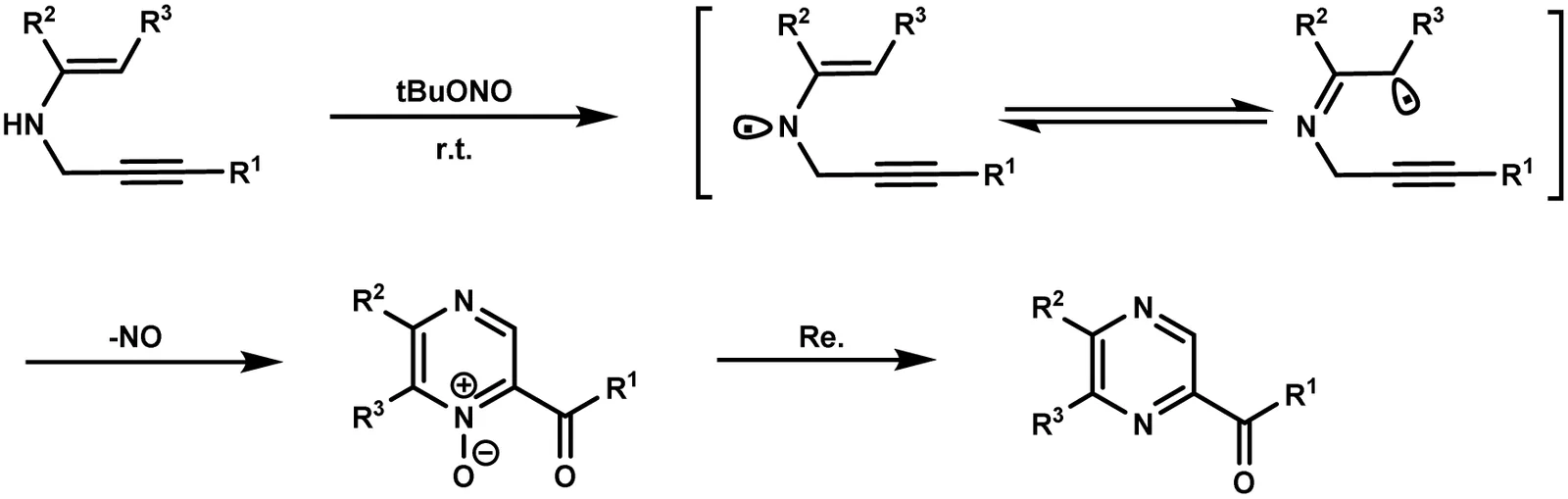
Metal-free [5 + 1] cyclisation to polysubstituted pyrazine 1-oxides (40–71% yield).

In 2020, Xia and co-workers reported a metal-free oxidative [5 + 1] cyclisation employing 1,5-enynes (0.2 mmol) and *tert*-butyl nitrite (TBN) under an O_2_ atmosphere at room temperature for 30 min.^67^ Through a cascade of NO radical incorporation, intramolecular radical cyclisation and insertion of an oxygen atom from water, polysubstituted pyrazine 1-oxides were obtained in 40–71% yield (successfully scaled to 2.0 mmol). This strategy accommodated aryl enynes bearing electron-donating or electron-withdrawing groups as well as heteroaromatic rings, and could be scaled up to 2 mmol. The products could be further transformed into pyrazines or ring-opened hydrolysis products (Scheme 28). Recent reviews have summarised advances in the construction of heterocyclic *N*-oxides *via* cyclisation strategies and in the visible-light-mediated functionalisation of heterocyclic *N*-oxides.^68,69^

The metal-free radical cyclisation strategy reported by Xia offers a conceptually distinct approach to pyrazine synthesis that lies outside the classical condensation–oxidation and cross-coupling paradigms. The reaction uses TBN as a radical source and O_2_ as the terminal oxidant, avoiding transition-metal catalysts, and proceeds at room temperature. However, the substrate scope is currently confined to 1,5-enynes, which are not as readily accessible as the simple diketones or ketones employed in other methods. The moderate yields (40–71%) and the requirement for specialised substrates suggest that this method is, at present, better suited to the synthesis of pyrazine *N*-oxide derivatives bearing specific substitution patterns than as a general tool for pyrazine synthesis. Nonetheless, the mechanistic interest and the potential for further expansion of the substrate scope warrant continued investigation.

### 5.6 Other novel cyclization strategies

**Scheme 29:**
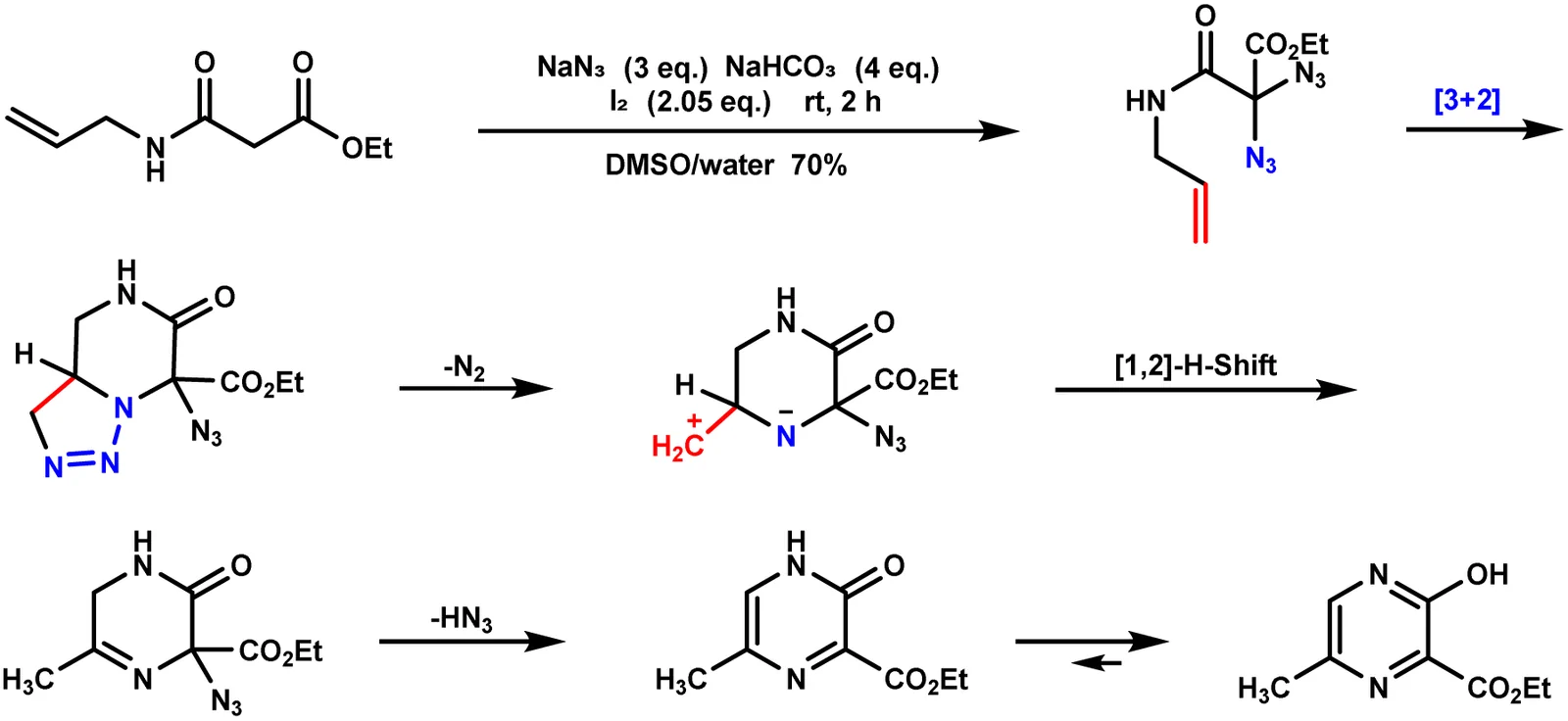
Cascade cyclization of *gem*-diazides to 2-hydroxy-3-esterpyrazines (43–74% yield).

In 2017, Ballaschk and co-workers reported a route starting from *N*-allyl malonamides. Diazidation with I_2_/NaN_3_ furnished geminal diazides, which upon thermolysis in xylene or heating with CuI/AcOH underwent a cascade of intramolecular 1,3-dipolar cycloaddition, nitrogen elimination, hydrogen migration and hydrazoic acid elimination to afford 2-hydroxy-3-ester-substituted pyrazines in 43–74% yield.^70^ The products could be further elaborated through *N*/*O*-alkylation, Suzuki coupling and side-chain bromination, offering modular access to functionalised derivatives (Scheme 29). The strategy of employing azides as radical acceptors in cascade cyclisations for the construction of N-heterocycles has been the subject of a recent review.^71^

### 5.7 Comparative summary

No single method is universally optimal; the choice depends on the target pyrazine structure, the desired scale and the available resources. A comparison of the major synthetic approaches is summarised in Table 3, while a decision framework for selecting methods based on target type is provided in Table 4.

**Table 3:** Comparison of major synthetic approaches for pyrazines

Method class	Typical conditions & yields	Substrate scope	Scalability	Advantages	Limitations
Classical condensation (α-aminoketone self-condensation)	α-Aminoketone; heat; 40–70%	Symmetrical 2,5-diaryl/dialkyl pyrazines	Lab scale	Mechanistic insights	Isomer mixtures; retro-Mannich fragmentation
Classical condensation (α-diketone + diamine)	α-Diketone + diamine; reflux; MnO_2_; 55–85%	2,3-Disubstituted pyrazines	Lab; industrial *via* gas-phase	Well-established	Two steps; stoichiometric MnO_2_ waste; side reactions
Industrial heterogeneous catalysis	Gas-phase dehydrocyclisation; Cu–Cr, Zn–Cr or Pt; 43–100%	Alkylpyrazines	Multi-tonne scale	High conversion/selectivity; continuous operation	High temperature; Cr toxicity; precious metal
Pd-catalysed cross-coupling	Halopyrazine + organometallic; Pd; 60–120 °C; 60–95%	Broad	Lab to pilot scale	Precise regiocontrol; late-stage diversification	Pre-functionalised substrates; precious metal; waste
Solvent-free grinding (Ghosh)	1,2-Diketone + diamine; RT grinding; 72–86%	2,3-Disubstituted pyrazines	Lab scale	No solvent/catalyst; air oxidation; operationally simple	Limited to simple 2,3-disubstituted pyrazines
Amino acid-based synthesis	Amino acid → pyrazine (*via* ester reduction or Dakin–West)	Symmetrical pyrazines	Lab to 100 g	Renewable feedstocks	Multistep; reagent intensive or cryogenic
Electrochemical oxidative coupling	Aryl methyl ketone + diamine; 43–81%	Monosubstituted pyrazines	Lab scale	No transition metals; no chemical oxidants	Energy input; Pt cathode
Metal-free radical cyclisation	1,5-Enyne + TBN; RT, O_2_; 40–71%	1,5-Enynes	Lab scale	Metal-free; mild	Specialised substrates
Method class	Typical conditions & yields	Substrate scope	Scalability	Advantages	Limitations

**Table 4:** Decision framework for selecting pyrazine synthetic methods

Target type	Key consideration	Recommended approaches	Limitations to consider
Simple symmetrical pyrazines (*e.g.*, 2,5-disubstituted)	Low cost, minimal waste, operational simplicity	Solvent-free grinding (Section 5.1)	Limited to symmetric products; poorly suited for complex targets
Biomass-derived pyrazines (*e.g.*, from amino acids or diols)	Renewable feedstocks; sustainability	Amino acid-based synthesis (Section 5.2); Pt/CeO_2_/Al_2_O_3_ (Section 5.4)	Stoichiometric reagents (Dakin–West); precious metal (Pt)
Complex or unsymmetrical targets	Precise regiocontrol; late-stage diversification	Pd-catalysed cross-coupling (Section 4)	Requires pre-functionalised substrates; relies on precious metal
*N*-Oxide or fused pyrazine derivatives	Access to specific scaffolds	Metal-free radical cyclisation (Section 5.5); electrocyclisation/cycloaddition (Section 4.1)	Moderate yields; specialised substrates

For simple symmetrical pyrazines, solvent-free grinding protocols (Section 5.1) offer a straightforward and low-waste approach, requiring neither solvents nor catalysts and proceeding at room temperature under air.

For biomass-derived pyrazines, amino acid-based syntheses (Section 5.2) enable the preparation of substituted pyrazines from renewable feedstocks, while the Pt/CeO_2_/Al_2_O_3_ catalyst (Section 5.4) directly converts biomass-derived diols into alkylpyrazines with catalyst recyclability.

For complex or unsymmetrical targets, palladium-catalysed cross-coupling (Section 4) remains widely used, although its larger reagent and energy footprint continues to motivate the search for alternative approaches. Among the emerging methods, electrochemical oxidation (Section 5.3) provides a transition-metal-free pathway, and metal-free radical cyclisation (Section 5.5) grants access to pyrazine *N*-oxide scaffolds.

It should be noted, however, that the scales at which these methods have been demonstrated vary considerably. The Dakin–West amino acid route has been conducted on a 100 g scale,^57^ and the electrochemical method has been scaled to 10 mmol.^60^ By contrast, most solvent-free, metal-free, and amino acid-based protocols have been reported at 0.2–1 mmol scale and should not be directly compared with continuous industrial vapour-phase processes without acknowledging this difference.

### 5.8 Summary

The period 2010–2020 witnessed a clear evolution of pyrazine synthesis towards protocols that reduce or eliminate solvents, catalysts, and stoichiometric oxidants—including solvent-free, catalyst-free, room-temperature and aerobic conditions—alongside broader feedstock diversification (from amino acids to simple ketones) and greater process intensification through electrochemistry and tandem reactions. Traditional condensation–oxidation routes were refined through solvent-free and catalyst-free variants; oxidative coupling and electrochemical methods that start directly from ketones avoided the need for pre-functionalised α-diketones; and amino-acid-based Dakin–West and DIBAL-H reduction routes utilised renewable feedstocks. Ballaschk's *gem*-diazide cascade further demonstrated an alternative cyclisation strategy for accessing functionalised pyrazine scaffolds.

Between 2020 and 2026, these trends continued. Natural product catalysis (onion extract) and heterogeneous catalysis (zeolites, Pt/CeO_2_/Al_2_O_3_, ZnAl mixed oxides) reduced the consumption of organic solvents and corrosive reagents, while offering catalyst recyclability. Metal-free radical cyclisation strategies provided additional routes to complex pyrazine frameworks. Collectively, these advances have not only enriched the synthetic toolbox for pyrazines but also opened possibilities for the preparation of bio-based pyrazines.

Despite these advances, challenges persist. The substrate scope of many of these methods remains narrower than that of palladium-catalysed cross-coupling, and the scalability of electrochemical and biocatalytic processes requires further development. The integration of emerging technologies—photocatalysis, flow chemistry and AI-assisted optimisation may offer further opportunities. The broader implications of biomass-derived routes to N-heterocycles such as pyrazines, have been thoroughly discussed in recent comprehensive reviews that cover both synthetic methodologies and biological applications.^51,72^

Across all methodologies, several persistent challenges remain. Regioselective access to unsymmetrical pyrazines continues to be a fundamental difficulty, whether through classical condensation or modern C–H functionalisation. Direct C–H functionalisation, while attractive, is still limited in substrate scope and regiocontrol compared with cross-coupling. The development of non-precious-metal alternatives remains an ongoing goal, as does improving functional-group compatibility and addressing catalyst deactivation in both homogeneous and heterogeneous systems. The purification of closely related regioisomers and the establishment of robust sustainability metrics—beyond simple qualitative labels—also require further attention. These challenges, together with the need for scalable continuous manufacturing, define the frontier of pyrazine synthesis methodology.

## 6. Conclusions and outlook

The chemical synthesis of pyrazines has undergone more than a century of development, evolving from the early foundational work on α-aminoketone self-condensation and α-diketone–diamine condensation, through the industrial applications of heterogeneous catalytic systems in the late 20th century, to the innovative strategies of the 21st century driven by green chemistry principles. A rich and mature synthetic toolbox has thus been established.

Looking back over this evolutionary journey, four clear threads can be discerned. First, the classical condensation–oxidation routes have been continuously simplified and greened, moving from high-temperature/high-pressure conditions to room-temperature, solvent-free protocols, and from stoichiometric oxidants to air oxidation. Second, transition metal-catalysed cross-coupling has enabled the precise late-stage functionalization of the pyrazine ring, offering unprecedented structural diversity for medicinal chemistry and materials science. Third, heterogeneous catalysis has continued to play a cornerstone role in industrial production, with copper-chromite, zinc-chromite and platinum-based catalysts achieving highly selective and high-yielding gas-phase syntheses of alkylpyrazines, while newer chromium-free and biomass-derived systems are addressing environmental concerns. Fourth, non-traditional strategies such as metal-free radical cyclisation and electrochemical oxidation have opened up new avenues for constructing complex pyrazine frameworks beyond classical condensation and coupling methodologies.

Looking forward, four specific unresolved synthetic challenges merit particular attention for future methodological innovation. First, the selective synthesis of unsymmetrical highly substituted pyrazines without prefunctionalisation remains a fundamental challenge, as classical condensation routes suffer from poor regiocontrol and cross-coupling methods require pre-functionalised halides. Second, the development of chromium-free continuous production processes is needed to replace the toxic Cu–Cr and Zn–Cr catalysts that still underpin much industrial pyrazine manufacture. Third, direct biomass-to-pyrazine conversion with high carbon efficiency remains an aspirational goal: although amino acid-based routes and Pt/CeO_2_/Al_2_O_3_-catalysed diol amination represent progress, they still rely on stoichiometric reagents or precious metals. Fourth, the translation of emerging electrochemical and continuous-flow methods from laboratory demonstrations to preparative or industrial scales requires further development of reactor engineering, electrode materials, and process stability.

Addressing these challenges will likely benefit from several enabling technologies. Machine learning has recently begun to find application in pyrazine chemistry; for example, Braconi and Godineau employed Bayesian optimization to optimize Cu-catalysed C–N coupling of sterically hindered bromo-pyrazines, identifying high-yielding conditions from a reaction space of over 138 000 possible combinations using 80 experiments, with the optimized protocol successfully scaled to 20 mmol.^76^ Photocatalytic transformations and biocatalytic routes, though still in their infancy, offer fundamentally different approaches to sustainable pyrazine synthesis.^73–79^ Beyond synthesis methodology, the exploration of pyrazine-based functional materials—including optoelectronics, covalent organic frameworks, and metal–organic frameworks—presents opportunities for synergistic development of synthetic chemistry and materials applications. We anticipate that continued advances in these directions will drive pyrazine synthesis towards more selective, sustainable, and scalable processes.

## Author contributions

Bingyang Wang and Yao Zhao contributed equally. Bingyang Wang: investigation, literature curation, formal analysis, writing – original draft; Yao Zhao: investigation, formal analysis literature curation, writing – original draft; Weihua Chen: investigation; Lianchen Zhao: literature curation; Qian Yang: investigation; Jie Ren: investigation, formal analysis; Guanglu Wang: investigation, formal analysis; Xiaoru Wang: supervision, writing – review & editing.

## Conflicts of interest

There are no conflicts of interest declared.

## Data Availability

In this review, no primary research results have been included, and no new data were generated or analysed. All the data used in this study are already published. All of the references are correctly cited within the manuscript.
